# Toward explainable AI (XAI) for mental health detection based on language behavior

**DOI:** 10.3389/fpsyt.2023.1219479

**Published:** 2023-12-07

**Authors:** Elma Kerz, Sourabh Zanwar, Yu Qiao, Daniel Wiechmann

**Affiliations:** ^1^Department of English and American Studies, RWTH Aachen University, Aachen, North Rhine-Westphalia, Germany; ^2^Institute for Logic, Language and Computation, University of Amsterdam, Amsterdam, Netherlands

**Keywords:** artificial intelligence, explainable AI (XAI), automated mental health detection, machine learning, deep learning, natural language processing, digital NLP-derived biomarkers, digital phenotyping

## Abstract

Advances in artificial intelligence (AI) in general and Natural Language Processing (NLP) in particular are paving the new way forward for the automated detection and prediction of mental health disorders among the population. Recent research in this area has prioritized predictive accuracy over model interpretability by relying on deep learning methods. However, prioritizing predictive accuracy over model interpretability can result in a lack of transparency in the decision-making process, which is critical in sensitive applications such as healthcare. There is thus a growing need for explainable AI (XAI) approaches to psychiatric diagnosis and prediction. The main aim of this work is to address a gap by conducting a systematic investigation of XAI approaches in the realm of automatic detection of mental disorders from language behavior leveraging textual data from social media. In pursuit of this aim, we perform extensive experiments to evaluate the balance between accuracy and interpretability across predictive mental health models. More specifically, we build BiLSTM models trained on a comprehensive set of human-interpretable features, encompassing syntactic complexity, lexical sophistication, readability, cohesion, stylistics, as well as topics and sentiment/emotions derived from lexicon-based dictionaries to capture multiple dimensions of language production. We conduct extensive feature ablation experiments to determine the most informative feature groups associated with specific mental health conditions. We juxtapose the performance of these models against a “black-box” domain-specific pretrained transformer adapted for mental health applications. To enhance the interpretability of the transformers models, we utilize a multi-task fusion learning framework infusing information from two relevant domains (emotion and personality traits). Moreover, we employ two distinct explanation techniques: the local interpretable model-agnostic explanations (LIME) method and a model-specific self-explaining method (AGRAD). These methods allow us to discern the specific categories of words that the information-infused models rely on when generating predictions. Our proposed approaches are evaluated on two public English benchmark datasets, subsuming five mental health conditions (attention-deficit/hyperactivity disorder, anxiety, bipolar disorder, depression and psychological stress).

## 1 Introduction

Mental illness, also known as mental disorders or psychiatric disorders, are increasingly prevalent worldwide and constitute one of the greatest challenges facing our healthcare systems and modern societies in general (1). This is demonstrated by the staggering number of 970 million people worldwide suffering from mental disorders, with anxiety and depression being the most common, costing the global economy over $1 trillion annually (World Health Organization 2019^1^). In fact, these disorders have a greater economic impact than cancer, cardiovascular diseases, diabetes, and respiratory diseases. The importance of mental health is widely recognized, as evidenced by its inclusion in the 17 United Nations Sustainable Development Goals (SDGs 2022^2^).

Given the profound impact of mental disorders, their prevention and regulation have become significant public health issues. By prioritizing mental health, we can enhance the overall well-being of individuals and society, while simultaneously mitigating the financial burden of these disorders. This underscores the need for a concerted effort to address mental health issues to promote well-being and improve the quality of life for affected individuals and society as a whole. In this context, early detection of mental illnesses is crucial for providing effective patient care and improving outcomes.

Mental health refers to a person's overall psychological and emotional wellbeing, encompassing the ability to cope with stress, achieve their potential, engage in fulfilling relationships, and contribute positively to society. Mental disorders are conditions that affect a person's mental health and can result in significant changes to their thoughts, feelings, behaviors, and overall functioning. These conditions can be caused by a combination of biological, environmental, and social factors, and may be characterized by a wide range of symptoms, such as changes in mood, behavior, or personality. Major mental disorders include depression, chronic stress and anxiety [see American Psychiatric Association (2), for the Diagnostic and Statistical Manual of Mental Disorders, DSM-5].

Unlike other chronic conditions, which rely on laboratory tests and measurements, conventional approaches to the assessment of mental health conditions rely on intermittent self-reports in response to standardized questionnaires, such as the Perceived Stress Scale (3) or the Depression Anxiety and Stress Scale (4). Although the validity of these methods have been established, they face two major drawbacks: delays in diagnosis associated with their use, which makes them unsuitable for early detection, and their subjectivity and dependence on individuals' memory and situational awareness. Moreover, such conventional approaches to identifying at-risk individuals are problematic because of social stigma and the unavailability of clinical psychologists (5). Another hurdle in conventional clinical assessment is the potentially episodic nature and high comorbidity of mental disorders [see, e.g., Diagnostic and Statistical Manual by the American Psychiatric Association (2) or ICD-11 by the WHO (6)]. This hurdle makes it difficult to separate overlapping symptoms into underlying discrete diagnoses, as reflected in the low inter-rater reliability and test-retest reliability for certain psychiatric diagnoses (7). For example, the clinical diagnosis of bipolar disorder is a protracted and costly process that takes an average of 10 years to complete (8). This prolonged diagnostic process may result in inadequate or inappropriate treatment, exacerbating the symptoms of bipolar disorder and increasing the risk of adverse outcomes (9).

Recent advances in Artificial Intelligence have stimulated research efforts toward developing more cost-effective and less intrusive methods. In particular, Natural Language Processing (NLP) in combination with Machine Learning (ML) is increasingly recognized as having transformative potential to support healthcare professionals and stakeholders in the early detection, treatment and prevention of mental disorders (see 10–12, for comprehensive reviews). The application of NLP and ML techniques facilitates the development of computational solutions for analyzing clinically relevant information on a large scale in Digital Psychiatry. This includes not only routinely collected data, such as Electronic Health Records (EHRs), but also patient-generated text or speech (13). Patient-generated content has been made available through social media platforms that are particularly appealing to the research community due to their scale and deep entrenchment within contemporary culture (14, 15). Individuals increasingly use social media to share their interests (16), personal daily life experiences (17), and even their moods and feelings (18). Unlike documentation created by healthcare providers, social media data presents individual thoughts, emotions, and dialogues in a personal voice. These data sources have gained significant importance in modeling and understanding public health concerns and has led to the emergence of a new field of research known as “Mental Illness Detection and Analysis on Social media”, henceforth MIDAS (see 19, for a recent review).

MIDAS is an interdisciplinary field situated at the intersection of multiple disciplines, including Computational Linguistics, Computational Social Science, Cognitive Psychology, and Clinical Psychiatry. Within this field, automated detection of mental health conditions is typically approached as a classification task or sentiment analysis, where NLP techniques are used to extract linguistic, statistical, and domain features from social media data. These features are then fed into supervised machine learning models to predict the presence of specific mental disorders and symptomatology. This approach holds significant potential for the development of a digital phenotype, a computationally derived characterization of an individual that can be analyzed for signs of mental illness, allowing for early detection and intervention (20–22).

Although the field of MIDAS has made significant progress, recent studies have prioritized predictive accuracy over model interpretability by relying on deep learning techniques and architectures, as noted by Su et al. (23), Zhang et al. (11), and Greco et al. (24). However, in sensitive applications such as healthcare, it is crucial to prioritize explainability, which refers to the ability to understand and interpret how a model reached its predictions. This is because incorrect predictions or recommendations in healthcare can have life-altering or life-threatening consequences. Consequently, research on Explainable AI (XAI) has gained significance, and there have been calls for the development of interpretable approaches in both general (25, 26) and medical-specific contexts (27–29).

In response to these calls, we aim to advance research on XAI in the realm of automatic detection of mental disorders from language behavior using textual data from social media. To achieve this aim, we conduct a systematic investigation of several XAI approaches, including global and local as well as model-intrinsic and model-agnostic explanation methods. Specifically, the major contributions of our work are as follows:

- We build separate binary classification models for each of the five mental health disorders under investigation: attention deficit hyperactivity disorder, anxiety, bipolar disorder, depression, and psychological stress. We train these models on a diverse range of interpretable features, surpassing the predominantly employed lexicon-based approaches found in current MIDAS literature. These features are capable of capturing various aspects of language usage, such as morpho-syntactic complexity, lexical sophistication and diversity, linguistic style, psychometric attributes, readability, and discourse cohesion.
- We move beyond the standard method of representing feature values in social media posts using aggregate statistics. Instead, we adopt the sliding-window technique to compute a sequence of measurements that capture the “local” distributions of feature values within each post. These distributions are then fed into a bidirectional long short-term memory (BiLSTM) network, which is capable of leveraging temporal information to make accurate predictions. By utilizing this approach, we aim to capture the dynamic changes in feature values within each post.
- We weigh the trade-off between interpretability and performance by comparing these BiLSTM models with a state-of-the-art pretrained language model (PLM).
- We conduct extensive feature ablation experiments to determine the most informative groups of interpretable features associated with specific mental health conditions.
- We present three significant advancements to a recently proposed approach that enhances the interpretability of PLM using an emotion-infused multi-task learning method. Firstly, we apply this approach to five different mental health conditions, expanding on its previous use for a single condition. Secondly, we connect the mental health detection task with not only emotion detection but also personality recognition. Lastly, we employ both model-agnostic and model-specific attention-based explanation techniques to identify the types of words that each PLM relies on for making predictions.

Our work addresses the following three research questions:

1. How do mental health prediction models, trained on interpretable features, compare with a state-of-the-art PLM that is domain adapted? What is the trade-off between interpretability and accuracy? (**RQ1**)
2. Which groups of linguistic features are most predictive of specific mental health conditions? (**RQ2**)
3. What insights can be gained into PLMs for mental health detection through the application of information-infused multi-task learning methods? (**RQ3**)

The remainder of this research article is structured as follows: Section 2 provides a concise overview of related work. Section 3 presents the datasets used and their respective descriptive statistics. Section 4 outlines the feature framework for text-based mental health detection. Section 5 describes the proposed mental health detection models. Section 6 describes the experimental setup used to evaluate the proposed models, along with the results obtained. Section 7 provides a discussion of the main findings. Finally, Section 8 summarizes the conclusions drawn from the work.

## 2 Related work

In this section, we provide a concise overview of the existing body of literature on Mental Illness Detection and Analysis on Social media (MIDAS). This overview serves as the backdrop for understanding of the main aim and methodologies employed in our work. For more comprehensive reviews of MIDAS research, readers may refer to Skaik and Inkpen (30), Chancellor and De Choudhury (31), Ríssola et al. (32), and Garg (19).

The field of MIDAS has developed innovative approaches to automatically label social media data, enabling the creation of supervised machine learning models that can detect various mental disorders. The approach by Coppersmith et al. (33) was a pioneering effort to identify public self-disclosures related to mental health using regular expressions. They tested this approach using Twitter data and collected a data set of 1,238 Twitter users with four mental health conditions (bipolar, depression, post traumatic stress disorder and seasonal affective disorder) using diagnosis statements such as “I was diagnosed with depression”. The dataset constructed in this work was utilized in subsequent MIDAS studies and has been featured at the CLPsych 2015 shared task (34). The approach of Coppersmith et al. (33) was then extended to data from the social media platform Reddit (35, 36). Unlike Twitter, Reddit allows for posts of unlimited length, thereby enabling the analysis of longer language samples that may carry more nuanced linguistic signals. Furthermore, Reddit is a more interactive, discussion-oriented forum that better mimics real-life communication. Reddit is structured into subreddits, which are dedicated forums focused on specific topics, including numerous subreddits pertaining to particular mental health conditions. This provides an opportunity to analyze mental health-related language within a more focused context and to gain insights that may not be evident from more general analyses.

Cohan et al. (36) made significant refinements to the original approach of Coppersmith et al. (33) by implementing several modifications. Firstly, the researchers developed detection patterns with high precision, which were based on diagnosis keywords obtained from the Diagnostic and Statistical Manual of Mental Disorders (DSM-5) headings. Secondly, the approach included the incorporation of synonyms, common misspellings, vernacular terms, and abbreviations to improve the accuracy of mental health signal detection. Thirdly, the researchers excluded potential diagnosed users who matched a negative diagnosis pattern (such as “I was never clinically diagnosed”) from the dataset. Fourthly, they removed any posts for diagnosed users and control users alike that contained any mental health terms (like “diagnosis”, “mental illness”, or “suffering from”) as well as any posts from the dataset that were made in any of the mental health-related subreddits. These refined modifications resulted in a carefully constructed mental health dataset, which is one of the two primary datasets used in our work (see Section 3 below).

Earlier studies on MIDAS have commonly utilized linguistic features derived from Linguistic Inquiry and Word Count (LIWC), or statistical features such as term frequency-inverse document frequency (TF-IDF) and n-grams. These features were then combined with traditional machine learning algorithms like support vector machines (SVM), Adaptive Boosting (AdaBoost), and Decision Trees to construct predictive models for a range of mental health conditions, such as post-traumatic stress disorder, depression, bipolar disorder, and seasonal affective disorder (33) and schizophrenia (37).

Subsequent MIDAS studies have increasingly relied on deep learning methods that allow to extract features automatically without feature engineering. These methods include the use of diverse embedding techniques, such as GloVe word embedding (38) and word2vec (39), as well as contextualized language representation, such as Bidirectional Encoder Representations from Transformers [BERT, (40)] or MentalBERT (41). They also include leveraging methods based on convolutional neural networks (CNN), recurrent neural networks (RNN) or hybrid-based methods [see (11) for a comprehensive recent review of all]. For example, Cohan et al. (36) used next to traditional machine learning classifiers (Logistic Regression, SVM and XGBoost) CNN and FastText (42) to develop binary classification models for the identification of individuals with mental health conditions. In another study, Murarka et al. (43) used RoBERTa [Robustly Optimized BERT Pretraining Approach, Liu et al. (44)] to build multi-class models to identify five mental health conditions from Reddit posts (ADHD, anxiety, bipolar disorder, depression, and PTSD).

As mentioned in Section 1, deep learning techniques have demonstrated remarkable performance in detecting mental health disorders by employing feature extraction and complex neural network structures. Despite their success, these methods are frequently considered opaque or “black boxes” due to their inability to provide explanations for their predictions, which is particularly crucial in healthcare. Although the importance of interpretability in deep learning methods for mental health detection is acknowledged in the literature, limited research has been conducted in this area. To the best of our knowledge, the only existing MIDAS papers are Song et al. (45), Sekulic and Strube (46) and Turcan et al. (47). In their approach, Song et al. (45) proposed a feature attention network that integrates four distinct indicators (namely, depressive symptoms, sentiments, ruminative thinking, and writing style) to effectively simulate the process of depression detection while enhancing the interpretability of the model. Specifically, to extract sentiment features, the authors leverage SentiWordNet to categorize words as positive, neutral, or negative, followed by encoding these categories into one-hot vectors using an RNN. The other three features are generated via basic neural networks and specialized linguistic tools. To further enhance the model's performance, an attention mechanism is also employed. Sekulic and Strube (46) utilized a hierarchical attention network to perform binary classification tasks and predict whether a user is afflicted with any of nine distinct mental disorders. The authors initialized the input layer with 300 dimensional GloVe word embedding. To evaluate the model's effectiveness, the authors analyzed the attention weights of word-level features relevant to classification, and thereby investigated the limitations of the model. Turcan et al. (47) investigated the effectiveness of emotion-infused multi-task learning models in stress detection. The authors experimented with three different architectures. The first architecture is an alternating multi-task model, in which two single-task models share the same BERT layers during training. The primary stress task was trained using the Dreaddit dataset (48), while the secondary emotion task was trained using the GoEmotions dataset (49). The second architecture is the classical multi-task model, in which two tasks are trained simultaneously using the same dataset. The authors used a fine-tuned emotion BERT model to predict the emotion labels for each stress post, and then trained a new BERT model using these predicted emotion labels and the ground truth stress label of Dreaddit. The third architecture is the fine-tuned model that employs a model fusion strategy for stress prediction by utilizing the fine-tuned emotion model. Our work builds upon the explainability approach introduced by Turcan et al. (47) by expanding it to include four more mental health conditions. Furthermore, we enhance the approach by integrating insights from personality recognition, which is another pertinent application domain (see Section 5 for more details).

## 3 Materials

The data used in our work are sourced from four publicly available English language datasets: (1) the Self-reported Mental Health Diagnoses (SHMD) (36), (2) Dreaddit (48), (3) GoEmotions (49), and (4) MBTI Kaggle (50). The SHMD and Dreaddit datasets are utilized as primary datasets to construct classification models for five types of mental health conditions, while GoEmotions and MBTI Kaggle serve as secondary datasets for constructing information-infused models.

The SHMD is a large dataset of social media posts from Reddit users with nine mental health conditions (MHCs) corresponding to branches in the Diagnostic and Statistical Manual of Mental Disorders (DSM-5), an authoritative taxonomy for psychiatric diagnoses (2). As outlined in Section 2, users were identified by constructing patterns for discovering self-reported diagnoses of the nine MHCs. For instance, if a user stated “I was officially diagnosed with depression last month”, they would be identified as having depression. That means that in the SHMD dataset, positive labels indicate that individuals have disclosed having received an actual diagnosis by mental health professionals. It is important to clarify that individuals did not self-report their own mental health disorders but rather disclosed that they were clinically diagnosed with the disorder(s).

For each condition, a seed list of diagnosis keywords was used, compiled from the corresponding DSM-5 headings and expanded by synonym mappings. To prevent that target labels can be easily inferred from the presence of MHC indicating words and phrases in the posts, all posts made to mental health-related subreddits or containing keywords related to a mental health statuses were removed from the diagnosed users' data. This approach ensured that the language analyzed in the dataset was not biased toward mental health discussions and reflected a broader range of topics. The original SHMD dataset comprises 20,406 diagnosed users and 335,952 matched controls, with an average of 161.2 posts per diagnosed user and 310 posts per control user. To mitigate concerns raised by recent review articles that suggest the presence of a relatively small number of unique individuals in datasets, which can limit the generalizability of models to platforms that are already demographically skewed (31, 51), we chose to down-sample the SMHD dataset such that each post was contributed by a distinct user. We also ensured that the selected posts were of comparable length. This approach was implemented for four of the nine targeted MHCs in the dataset [attention deficit hyperactivity disorder (ADHD), anxiety, bipolar, and depression]. By doing so, we aimed to improve the diversity of individuals represented in the dataset and enable better generalization of models to other platforms.

Dreaddit is a dataset for the identification of psychological stress that comprises a total of 3.5k posts collected from categories of subreddits where members are likely to discuss stressful topics, such as interpersonal conflict, mental illness and financial need. The class for stress and non-stress labels were obtained by way of human annotation using Amazon Mechanical Turk. Each post was rated for the presence or absence of stress by five crowd-workers with final binary labels being obtained by way of majority vote. The annotations resulted in 47.75% of the posts being labeled as non-stress and 52.75% being labeled as stress.

Both SHMD and Dreaddit are considered a valuable resource for researchers studying mental health, as it provides a large and diverse sample of individuals discussing their experiences with mental health conditions in an informal online setting. These data sets are among the largest and most comprehensive resources available for researchers studying mental health, providing a diverse sample of individuals discussing their experiences with mental health conditions in an informal online setting. Based on these two data sets, we constructed a corpus with the goal of obtaining sub-corpora of equal size for the five MHCs targeted in this work. The five MHCs and the number of users in each condition, as well as the average length of posts (in terms of words and characters) for the positive and control groups are presented in Table 1.

**Table 1:** Total number of users/posts along with average post length (in words and characters) for the positive and control groups.

**Dataset**	**Classes**	**Total users**	**Avg. no of words**	**Avg. no of characters**
SMHD-ADHD	ADHD	5272	117.98	638.60
	Control	5270	97.83	527.88
SMHD-Anxiety	Anxiety	4963	116.45	619.73
	Control	4963	96.64	523.36
SMHD-Bipolar	Bipolar	3632	116.56	622.31
	Control	3632	97.27	524.08
SMHD-Depression	Depression	7818	114.70	610.82
	Control	7816	96.29	521.25
Dreaddit	Stress	1857	93.00	459.31
	Control	1696	85.50	434.91

GoEmotions is currently the largest available dataset that is manually annotated and available for emotion prediction. It comprises 58,000 Reddit comments, which are labeled by 80 human raters for 27 different emotion categories, including a neutral category. The authors have also provided a mapping of these 27 categories to Ekman's six fundamental emotions: anger, disgust, fear, joy, sadness, and surprise. These six emotions are assumed to be physiologically distinct (52, 53). Based on the findings from experiments with various emotion mappings as reported in Turcan et al. (47), the present work employs these six fundamental emotions.

The Kaggle MBTI dataset comprises social media interactions from 8,675 users who have indicated their Myers-Briggs Type Indicator (MBTI) personality type. This dataset was collected through the PersonalityCafe forum,^3^ which provides a diverse sample of people interacting in an informal online social environment. The MBTI questionnaire is a well-established tool that describes personality in terms of 16 types resulting from combining binary categories from four dimensions: (a) Extraversion/Introversion (E/I) - preference for how people direct and receive their energy, based on the external or internal world, (b) Sensing/Intuition (S/N) - preference for how people take in information, through the five senses or through interpretation and meanings, (c) Thinking/Feeling (T/F) - preference for how people make decisions, relying on logic or emotion over people and particular circumstances, and (d) Judgment/Perception (J/P) - how people deal with the world, by ordering it or remaining open to new information.

## 4 A feature framework for text-based mental health detection

In this section, we present a feature framework aimed at providing guidance in the organization and characterization of features for text-based detection and analysis of mental health conditions. A comprehensive set of 498 features examined in our work falls into two broad categories: (1) general linguistic features (GLFs) and (2) lexicon-based features (LBFs). GLFs encompass 192 features that fall into five distinct groups pertaining to multiple levels of linguistic organization, whereas LBFs encompass 306 features derived from seven lexicons. Our comprehensive set of GLFs draws on an interdisciplinary body of research that encompasses psycholinguistics, sociolinguistics, register/genre variation, human language learning and processing, text readability, and clinical psychiatry. The LBF category encompasses words and phrases from seven lexicons that have been successfully used in the field of mental illness detection or in related fields such as personality recognition, emotion recognition, and sentiment analysis. In sum, our feature framework leverages insights from a range of fields to develop a more nuanced understanding of language use and its relationship to mental health conditions.

GLFs are categorized into five distinct groups that span across multiple levels of linguistic organization, comprising (1) morpho-syntactic complexity, (2) lexical richness/complexity, (3) readability, (4) cohesion, and (5) stylistics. The measures of the first category of GLFs pertains to various aspects of **morpho-syntactic complexity** and encompass five distinct types: (a) length of production unit, (b) sentence complexity, (c) subordination, (d) coordination, and (e) particular structures. The second category of GLFs revolves around the multi-dimensional construct of **lexical richness** (also known as **lexical complexity**) that pertains to the variety and diversity of the vocabulary used in a language sample or text. The lexical measures investigated here fall into four types: (a) lexical diversity, (b) lexical sophistication, (c) lexical density and (d) word prevalence. The third category of GLFs, **readability**, relates to the ease with which a language sample can be read and understood. Measures of readability, such as the Flesch-Kincaid Grade Level, assess factors such as sentence length, word choice, and grammatical complexity to estimate the educational level required to understand a given text. In this work, a total of thirteen measures of readability are taken into account. The fourth category of GLFs, **cohesion**, relates to the explicit and implicit cues that facilitate connections between ideas presented in a language sample. Specifically, we have focused on two types of cohesion: (a) lexical overlap and (b) the use of connectives. The fifth group of GLFs, **stylistics**, focuses on language usage variations in terms of register, genre, and style. The measures in this group are operationalized as register/genre-specific n-gram measures and take into account the frequency rank and count of a given n-gram across various registers/genres, providing a more differentiated assessment of n-gram use. A more detailed overview and description of the GLFs can be found in Section 1 of the Supplementary material and in Supplementary Table S1.

As mentioned earlier, prior research in MIDAS has primarily relied on lexicon-based features (LBFs) to construct predictive models of mental health, with a focus on identifying the emotions, sentiments, and affects conveyed through written text. In our work, we utilized a total of seven lexicons:

1. The Affective Norms for English Words (**ANEW**) (54) comprises six affective norms for valence, arousal, dominance, and pleasure.
2. The **ANEW-Emo** (55), provides a discrete categorical characterization of ANEW based on collected ratings of the set on happiness, sadness, fear, disgust, and anger.
3. The General Inquirer (**GI**) (56) is a comprehensive dictionary that contains lists of words and phrases, classified according to their semantic and syntactic properties, and assigned to different categories such as positive and negative affect, strong and weak activity, and political, economic, and social terms.
4. The Geneva Affect Label Coder (**GALC**) (57) includes word lists associated with 36 specific emotions, including anger, guilt, joy, and hope, as well as two general emotional states.
5. The Linguistic Inquiry and Word Count (**LIWC**) (58) contains a range of categories to measure the frequency of words that reflect certain psychological and linguistic constructs. The LIWC lexicon consists of two main parts: the internal dictionary, which contains the actual word categories, and the external dictionary, which contains user-defined categories. The internal dictionary includes categories such as positive and negative emotions, social processes (e.g., communication, social words), cognitive processes (e.g., insight, certainty), and linguistic dimensions (e.g., pronouns, prepositions). Each word in the LIWC lexicon is assigned to one or more of these categories based on its meaning and usage in language.
6. The **EmoLex** (59) provides lists of words and bigrams evoking particular emotions (such as joy, sadness, anger, fear, or disgust).
7. The **SenticNet 5.0** (60) is a database that consists of a large set of concepts and associated polarity scores, representing the sentiment meanings of words and phrases.

**Figure 1:**
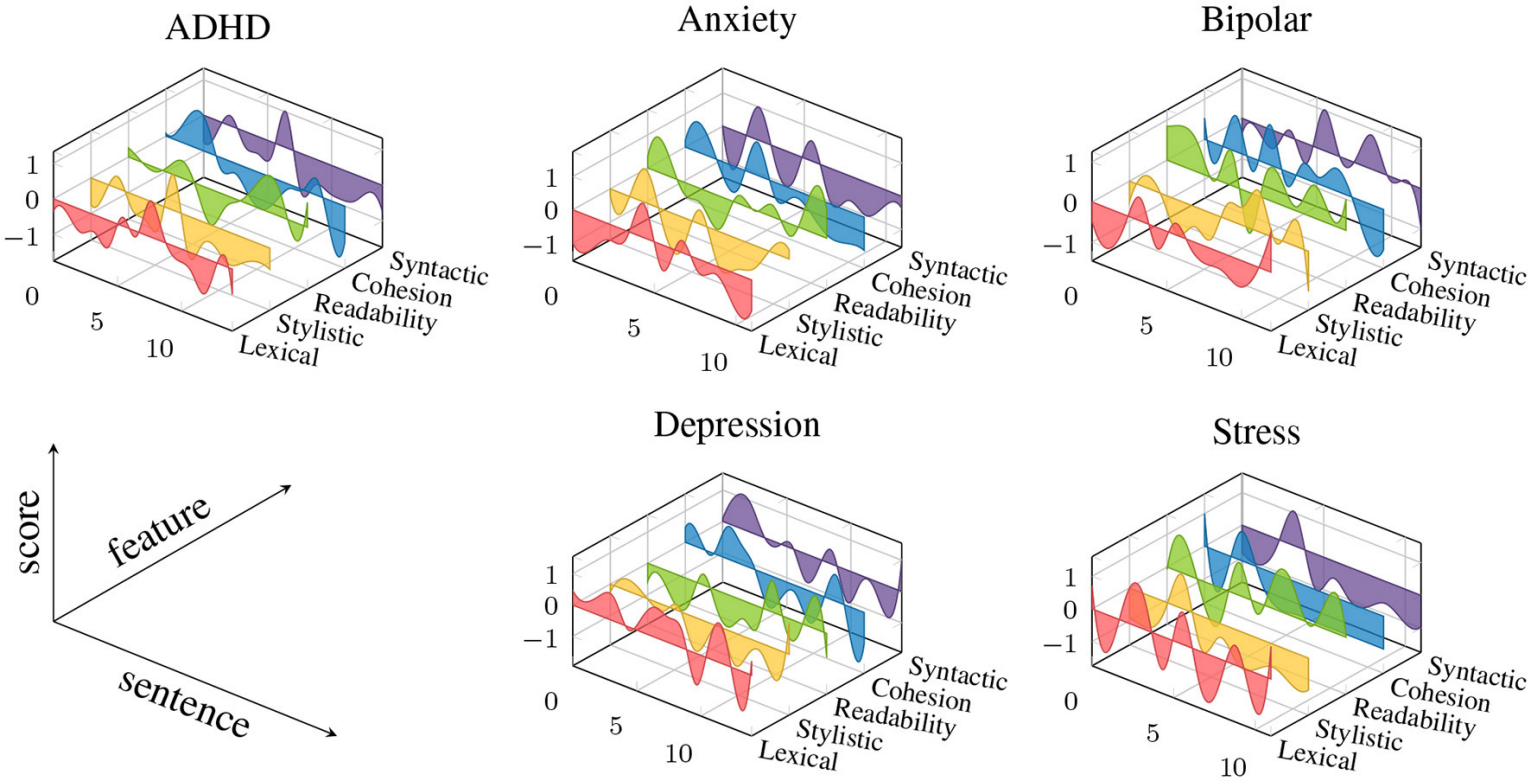
Examples of text contours from five texts representing the mental health conditions investigated in this work. The colored graphs represent the within-text fluctuations of feature values for five selected features representing each of the five groups of General Linguistic Features (GLF) (Cohesion: Overlap of words across adjacent sentences, Lexical: mean length of word (in characters), Readability: Flesch Kincaid Index, Stylistic: Bigram frequency score obtained from “weblog” register of Corpus of Contemporary American English, Syntactic: Mean length of sentence). All features scores are z-standardized and smoothed using b-spline.

To calculate the measurements for GLFs and LBFs, an automatic text analysis (ATA) system was used. This ATA system used employs a sliding-window technique to calculate within-text distributions of measurement values. This approach differs from the standard approach used in other ATA tools, which rely on aggregate scores representing the average value of a measure in a text. With the sliding-window technique, a window is moved across a text sentence-by-sentence, computing one value per window for a given measure of language performance. The ATA system utilizes the Stanford CoreNLP toolkit as an integral component for text pre-processing, including tokenization, sentence splitting, part-of-speech tagging, lemmatization, and syntactic parsing (61). The outputs of this toolkit are then passed to the measurement module to calculate scores for each individual text. The system generates a series of measurements representing the ‘local' distributions of values, which capture within-text distributions for each of general linguistic and lexicon-based features. These text contours are illustrated in Figure 1 for five selected features and five texts representing mental health conditions under investigation. The subsequent section describes how predictive models are trained on these in-text distributions. In Section 2 of the Supplementary material, we report preparatory analyses aimed at uncovering the statistical patterns of language use that distinguish the five mental health conditions we studied from the control groups.

## 5 Mental health classification models

**Figure 2:**
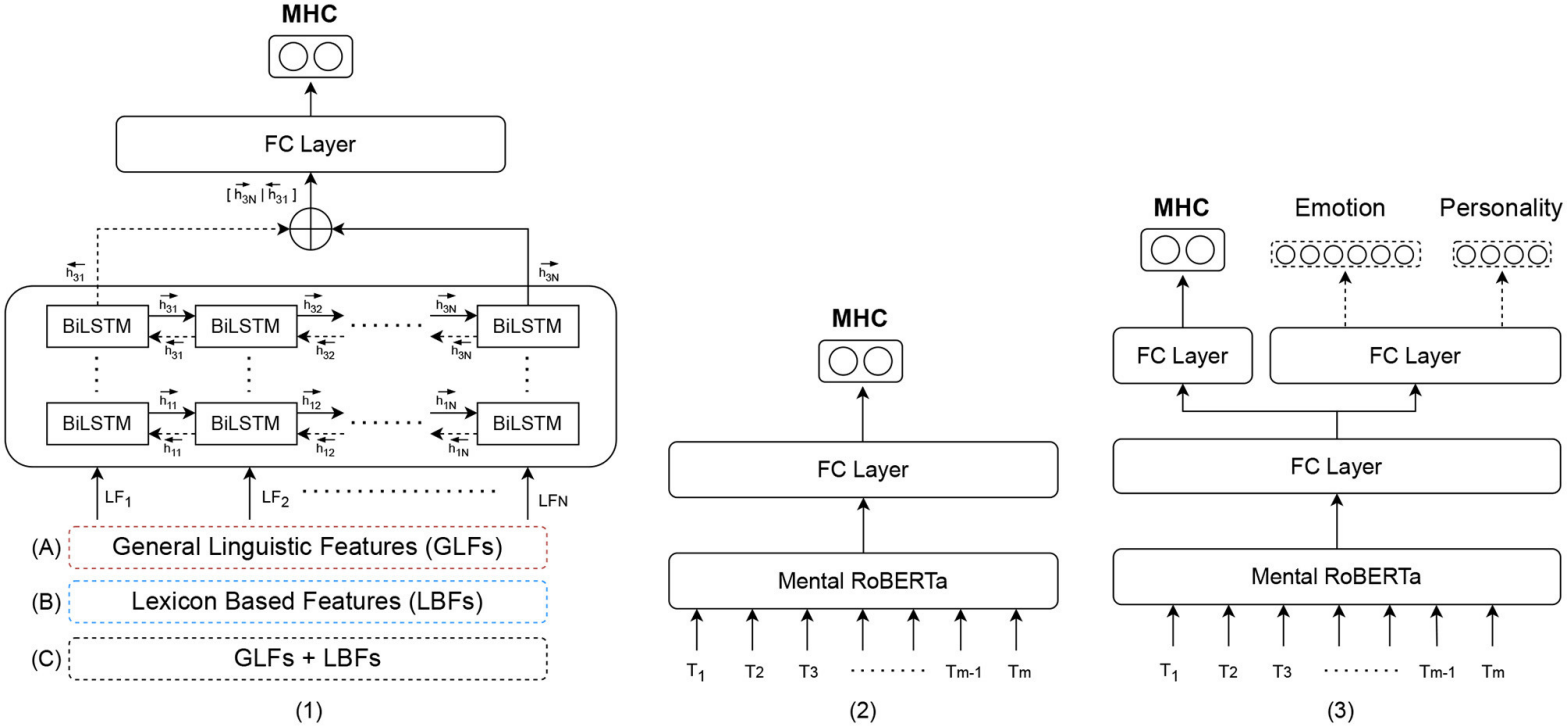
Schematic representation of the three model types for mental health detection: **Type 1**: (A) Bidirectional LSTM (BiLSTM) trained on general linguistic features (BiLSTM + GLFs), (B) BiLSTM trained on lexicon-based features (BiLSTM + LBFs) and (C) BiLSTM trained on the combination of GLFs + LBFs; **Type 2**: Pre-trained fine-tuned MentalRoBERTA; **Type 3**: Multi Task-Fusion Models: (A) Emotion-Infused Model, (B) Personality-Infused Model and (C) Emotion-Personality-Infused Model.

We build a series of binary classification models to predict five mental health conditions (anxiety, ADHD, depression, bipolar disorder and psychological stress). More specifically, we conduct extensive experiments with three types of models to weigh the trade-off between interpretability and classification performance, including its sub-types. The first type of model (**Type 1**) consists of BiLSTM (Bidirectional Long-Short Term Memory) models trained on interpretable features. Within this type, we construct three subtypes of models: (a) a BiLSTM trained on general linguistic features (BiLSTM + GLFs), (b) a BiLSTM trained on lexicon-based features (BiLSTM + LBFs), and (c) a BiLSTM trained on a combination of both groups of features (BiLSTM + GLFs + LBFs). The second type of models (**Type 2**) is (d) a state-of-the-art pre-trained and fine-tuned transformer, MentalRoBERTa (41). This PLM is a domain-specific language model trained on mental health data from the social platform Reddit. The third type of models (**Type 3**) encompasses task-fusion models: (A) Emotion-Infused Model, (B) Personality-Infused Model, and (C) Emotion-Personality-Infused Model. Figure 2 illustrates a schematic representation of the three main model types and their subtypes. As a result, we test and evaluate a total of 35 binary classification models. For more information on these models, see Subsections 5.1-5.3.

### 5.1 Bi-directional LSTM models trained on interpretable features

Recurrent neural networks (RNNs) are widely used due to their ability to process variable-length sequential data. RNNs have a general graph structure with cycles. These cycles allow activations of the previous time step to pass back into the network and thus capture long-range dependencies in the data. However, RNNs are generally difficult to train due to the so called exploding or vanishing gradients problem, which hinders the learning of distant dependencies within a longer sequence (62, 63). Long Short-Term Memory (LSTM; 62) neural networks are an extension of RNN that has been proposed to solve this problem. The idea of using LSTM networksis based on the approach that humans have the ability to retain memory over a short period of time.

The models trained only on interpretable features are constructed as 3-layer BiLSTMs with a hidden state dimension of 512.

From the output of the BiLSTM we choose the last hidden states in forward ($\vec{h_{n}}$) and backward directions ($\overset{⃖}{h_{n}}$), where *n* is the final layer of BiLSTM, in our case n=3. To predict the labels of a sequence, we concatenate the last hidden states of the last layer in forward and backward direction. The resulting vector $h_{3}=\left[\vec{h_{3}}|\overset{⃖}{h_{3}}\right]$ is then passed through a 2-layer feedforward neural network, whose activation function is Rectifier Linear Unit (ReLU). This is then fed to a final fully connected layer. The output for each text is obtained from this layer by applying a sigmoid function.

**Figure 3:**
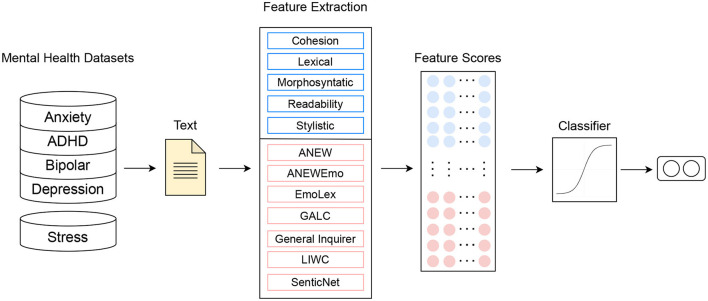
**Type 1 Models**: BiLSTMs trained on interpretable features.

**Figure 4:**
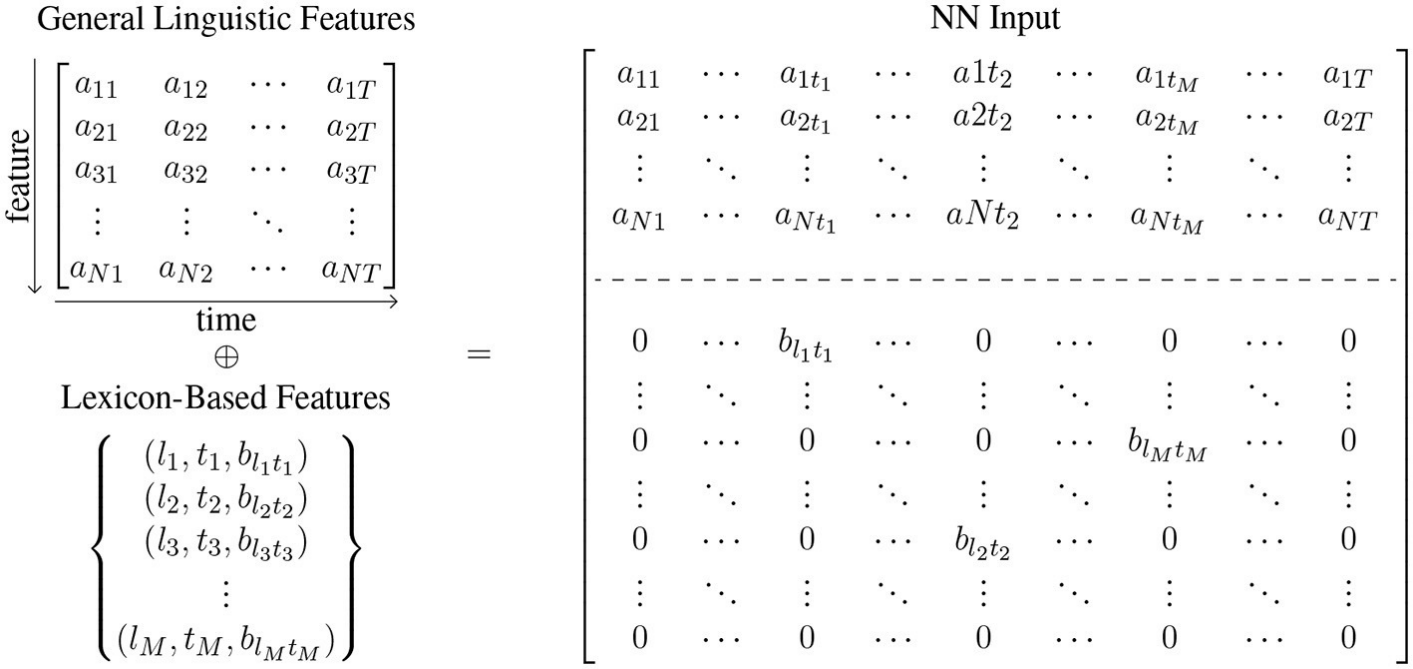
The values of the interpretable features that serve as input of the BiLSTM-based models are extracted by the automatic text analysis (ATA) system. The ATA system distinguishes between general language features and lexicon-based features. General linguistic features tend to result in a dense matrix, where *a*_*ji*_ is the feature score of *j*th general language feature for *s*_*i*_. In contrast, lexicon-based features tend to result in a sparse matrix, which is presented here as a set of 3-tuples (*feature id, sentence id, feature score*). A tuple (*j, i, b*_*ji*_) is included in the set, if and only if the *j*th lexicon-based feature yields a non-zero feature score *b*_*ji*_ for sentence *s*_*i*_.

On the basis of this general model architecture, we train three sub-types of models: (1) a model with general language features (BiLSTM + GLFs), (2) a model with lexicon-based features (BiLSTM + LBFs), and a model with all interpretable features (BiLSTM + GLFs + LBFs) (see Figure 3). The input of the BiLSTM models comprises the measurements of the interpretable features in a given text, assembled in a dense matrix format. Each cell in this matrix represents the value of a particular feature for a particular sentence. Consider a general language feature set $F_{G}=\left\{f_{1}^{\left(g\right)},f_{2}^{\left(g\right)},\ldots ,f_{N}^{\left(g\right)}\right\}$ of size *N* and a set of lexicon-based feature $F_{L}=\left\{f_{1}^{\left(l\right)},f_{2}^{\left(l\right)},\ldots ,f_{K}^{\left(l\right)}\right\}$ of size *K*. Given a text containing *T* sentences $s_{1}^{T}=\left\{s_{1},s_{2},\ldots ,s_{T}\right\}$, for each *s*_*i*_ the associated general language feature vector and associated lexicon-based feature are extracted. The lexicon-based feature vector for sentence is presented in Figure 4 as ${\left(a_{1i},a_{2i},\ldots ,a_{Ni}\right)}^{T}$, where *a*_*ji*_ is the feature score of $f_{j}^{\left(g\right)}$ for *s*_*i*_. Since lexicon-based features tend to result in a sparse matrix, we represent the feature matrix resulting from lexicon-based feature set in Figure 4 as a set of 3-tuples (*feature id, sentence id, feature score*). A tuple (*j, i, b*_*ji*_) is included in the set, if and only if a lexicon-based feature $f_{j}^{\left(l\right)}$ yields a non-zero feature score *b*_*ji*_ for sentence *s*_*i*_. In the case of the BiLSTM + GLFs + LBFs model the matrices from general linguistic and lexicon-based feature sets are concatenatedalong feature axis (see Figure 4). These matrices are then used as input to a BiLSTM. We chose the BiLSTM to learn the temporal information in the progression of the feature values within a text, i.e., to account for the changes in the features over time.

### 5.2 Pre-trained language models for mental healthcare (MentalRoBERTa)

**Figure 5:**
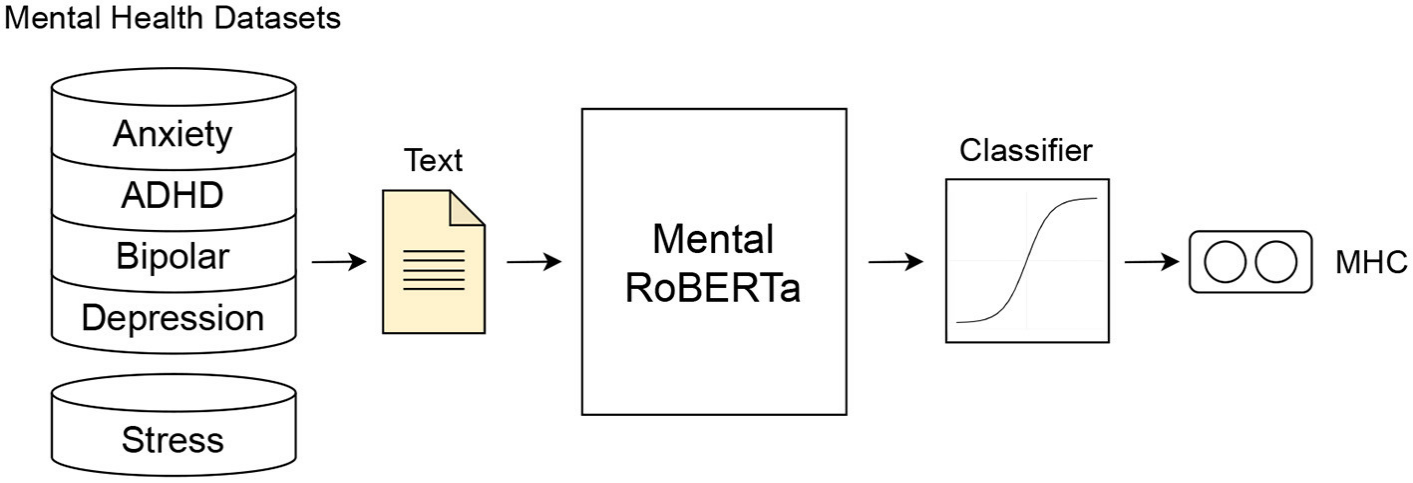
**Type 2 Model**: Fine-tuned Mental RoBERTa model.

As mentioned above, the Type 2 model is MentalRoBERTa, a state-of-the-art PLM for the domain of mental health (41). This PLM serves as a strong black-box baseline model, and it leverages domain-specific knowledge acquired through pre-training on a large-scale corpus of mental health-related text. This enables the model to effectively capture the subtleties and complexities of mental health language. A schematic representation of the model architecture can be seen in Figure 5. We use the MentalRoBERTa model, leveraging Hugging Face's Transformers library and the PyTorch framework. The base model design consists of 12 transformer layers with 12 attention heads and its hidden representation has a dimensionality of 768. To tokenize the sequences, the RoBERTa tokenizer was utilized. The input texts that are fed into the model have a maximum length of 512 words, since this is the highest number of tokens that BERT-based models can handle. From this we obtain an output of 768 dimensional vectors, which are then passed to a fully connected linear layer and then to a sigmoid function. The output is the probability that the text belongs to the positive label class. For the training we used a learning rate of 2*e*−5, weight decay of 1*e*−5 and batch size of 32 and trained the models for 8 epochs keeping the model with highest accuracy on the respective validation set.

### 5.3 Multi-task fusion models

Task fusion is a technique used in deep learning that involves the integration of multiple tasks within a single architecture, allowing for simultaneous learning. This approach enables fusion models to leverage cross-task information to improve performance on the primary task. In our case, we have developed multi-task fusion models for mental health prediction, which incorporate emotion and personality detection as secondary tasks. Emotion and personality are related constructs that may provide valuable information for understanding an individual's mental well-being. In our models, the primary task is to predict the mental health condition of an individual, such as depression or anxiety. The secondary tasks involve predicting the individual's emotional states and personality traits. By learning these secondary tasks simultaneously with the primary task, the model can leverage the cross-task information to improve its performance in predicting the mental health condition of the individual.

**Figure 6:**
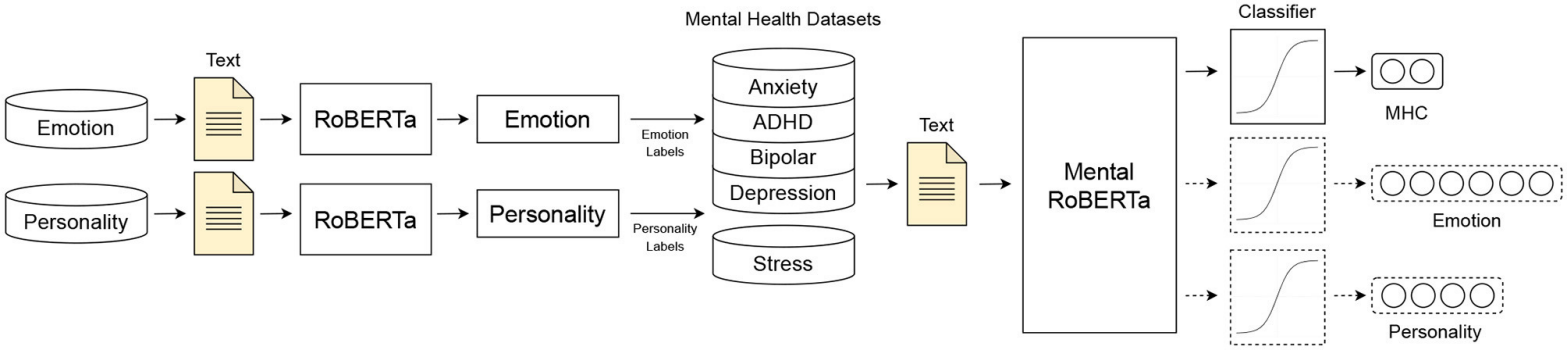
**Type 3 Models**: Multi-task Fusion models.

Our task fusion approach builds on the multi-task learning setup introduced by Turcan et al. (47). To construct emotion and personality labels for the two datasets, we used the method described in this work, as the SMHD data only provides labels for mental health conditions and Dreaddit only provides labels for stress. First, we independently trained RoBERTa models on the Kaggle MBTI and GoEmotions datasets and used them to produce “silver labels” for emotion and personality. Next, we trained our multi-task fusion model on two or all three tasks (mental health detection, emotion recognition, and/or personality detection) using the same training routine and parameters as the MentalRoBERTa model. Figure 6 illustrates the architecture of our task fusion models. In each multi-task fusion model, the loss is a weighted sum of the loss (𝓛tot) from the mental health detection task and the secondary task, with a tunable weight parameter λ*MHC*.


$$\begin{array}{l} L_{tot}=\lambda_{MHC}{𝓛}_{MHC}+\left(1-\lambda_{MHC}\right)L_{SEC} \end{array}$$

## 6 Evaluation

### 6.1 Methodology

To assess the models' performance in detecting the five mental health conditions, we employ four evaluation metrics: *accuracy*, *precision*, *recall* and *F*_1_. To assess model explainability, we utilize a combination of global and local, self-explaining, and *post-hoc* (aka model-intrinsic and model-agnostic) explanations methods (see, 64, 65). The distinction between local and global explanations concerns the level at which the explanation is situated. A local explanation justifies the model's prediction for a specific input, while a global explanation provides a justification that works independently of any particular input, such as quantifying feature importance. The distinction between self-explaining and *post-hoc* methods concerns whether explanations arise as part of the prediction process or require additional processing after the model has made a prediction. Self-explaining methods integrate the explanation process into the model's internal structure or the process of computing the output. For architectures with an attention mechanism (66), self-explaining methods can be derived from the relative attention values for each token (67, 68). In contrast, model-agnostic feature assignment methods utilize surrogate models after applying the predictive model. The most widely used method in this category is the “**l**ocal **i**nterpretable **m**odel-agnostic **e**xplanations”-method (LIME, 69). LIME generates perturbations by omitting subsets of tokens from the input text and then fitting a linear classifier to these local perturbations.

We perform three different analyses to probe our trained models and discover what information they learn to use. These analyses draw on both global and local explanation methods: First, for the Type 1 models trained exclusively on interpretable features, we perform feature ablation experiments based on a global explanation method. Second, for the Type 2 transformer-baseline model, we first quantify the reliance of a model on individual words during MHC detection using two local explanation methods. We then map the most important words to the categories of the hand-crafted LIWC-dictionary (see Section 4) to relate individual words to psychologically significant concepts to determine which types of words influence the prediction of a particular mental health condition. And third, we apply the same method to the Type 3 (task fusion) models to investigate the adjustment of word feature importance that results from enriching the models with information about the emotional state and/or personality trait of the author of the post. Further details of these global and local explanation methods are provided below.

The feature ablation experiments performed to better understand the prediction of the models based solely on interpretable features, we employ Submodular Pick Lime (SP-LIME) (69). SP-LIME is a method to construct a global explanation of a model by aggregating the weights of linear models that locally approximate the original model. To this end, we first construct local explanations using LIME. Separate analyses are performed for models trained on the general language features (GLFs) and those trained on the lexicon-based features (LBFs). The GLFs are divided into five groups (cohesion, lexical, morpho-syntactic, stylistic, and readability features), and importance scores are calculated for each of these groups. The LBFs are grouped according to the specific dictionary to which they belong, and importance scores are calculated for each dictionary. For more details, see Section 3 of the Supplementary material.

The explanation methods used to examine the transformer-based models are based on feature importance scores, i.e., feature attributions at the level of individual words (unigrams): For each social media post in the test set, we identify the 10 unigrams with the highest feature importance in the MHC prediction. In this way, we obtain 10 * *N*_*testset*_ individual unigram explanations for analysis, where *N*_*testset*_ denotes the number of classification instances in each test set.

We derive these feature importance scores using two local explanation methods: (i) a model-agnostic, *post hoc* method LIME (69) and (ii) a model-specific, self-explaining method based on attention gradients [AGRAD, Liu et al. (68)]. LIME takes an input from our testset, perturbs it in the bag-of-unigrams space, and runs the classification model on each perturbation to calculate the importance of each unigram. AGRAD measures whether a contextual embedding makes a positive or negative contribution to the results of a model, which is referred to as “importance discrimination”. The central idea behind importance discrimination is to calculate the gradient of loss with respect to attentional weights: A negative gradient indicates that a contextual embedding reduces the loss, whereas a positive gradient indicates that it increases the loss [see equation (2)], where *L* denotes the loss and α denotes the attention weight.


(1) $$\begin{array}{l} A_{i}=-\frac{\partial L}{\partial \alpha_{i}}\times \alpha_{i} \end{array}$$

Finally, to gain further insight into the effects of emotion and personality infusion for mental health detection and to discover what information the transformer models learn to use, we examine the differences in feature attribution between the Type 2 and Type 3 models. We quantify the differences between the models in terms of differences in word reliance resulting from the infusion of information pertaining to emotion, personality information or both.

### 6.2 Results

In the first step, we report on the results from a systematic comparison between Bi-Directional LSTM models trained on interpretable features and a transformer-based state-of-the-art model. Next, we present the results of our feature ablation experiments, which aimed to identify the most predictive type of interpretable features within the two primary groups: (1) general linguistic features (GLFs) and (2) lexicon-based features (LBFs). The third step involves presenting the outcomes of our multi-task fusion models, revealing the information they learn from the two associated domains of emotion detection and personality recognition. Finally, we present the results of two explanation techniques, namely the “local interpretable model-agnostic explanations” (LIME) method and a model-specific self-explaining method (AGRAD), in the fifth and final step.

The performance of twenty binary classification models is summarized in Table 2, with evaluation metrics including accuracy, precision, recall, and F1-score. The F1-score evaluation of three sub-type models belonging to the Type 1 category reveals the following results: The Type 1 models demonstrate a performance range of 57.14% to 70.78%, with the highest performance observed in detecting stress and the lowest in detecting ADHD. The F1-scores for detecting depression, anxiety, and bipolar disorder are similar, averaging around 64%. Among the five MHCs evaluated, the sub-type model C, which is a BiLSTM model trained on the complete set of interpretable general linguistic and lexicon-based features, achieves the highest F1 scores for detecting ADHD, depression, and psychological stress. These results indicate that the general linguistic and lexicon-based features are complementary to each other for detecting these three conditions. The subtype A model, which is a BiLSTM model trained exclusively on general linguistic features, achieves the highest F1 score for bipolar disorder. Conversely, the subtype B model, which utilizes a BiLSTM model trained solely on lexicon-based features, achieves the highest F1 score for anxiety. Our Type 1 models demonstrate superior performance compared to the existing benchmarks reported in Cohan et al. (36) original work. Specifically, we observed relative performance improvements of 11.47%, 10.48%, 9.33%, and 13.17% for ADHD, anxiety, bipolar, and depression, respectively. It is noteworthy that our BiLSTM models achieved such performance improvements despite being trained on only one post per user, whereas the models presented in Cohan et al. (36) are trained on an average of over 150 posts per user. Evaluation of the Type 2 models (MentalRoberta) using F1-score across the five conditions indicates that the highest performance is achieved for stress (F1=81.62%), followed by bipolar (F1=71.83%) anxiety (F1=70.5%), depression (F1=70.08%) and ADHD (F1=64.72%). The performance evaluation of the MentalRoBERTa model for stress detection shows a 2.74% improvement in F1-score compared to the BERT-based benchmark model presented in Turcan et al. (47) study. The comparison of the Type 1 models with the Type 2 models shows the difference in F1-scores ranging between 5.69% and 10.84%. The discrepancy in F1-scores is greatest for stress (-10.84%), followed by bipolar disorder (-7.64%) and depression (-7.19%). ADHD shows a difference of -6.15%. For anxiety, the discrepancy is smallest at -5.69%.

**Table 2:** Performance comparison between binary classification models: **(A)** BiLSTM trained on General Linguistic Features (GLFs), **(B)** BiLSTM trained on Lexicon-Based Features (LBFs), **(C)** BiLSTM trained on the combination of GLFs and LBFs, and **(D)** Pre-Trained Transformer-Based Language Model (Mental RoBERTa).

**Model**	**MHC**	**Accuracy**	**F1-score**	**Precision**	**Recall**
	ADHD	57.57	57.14	57.88	57.59
	Anxiety	62.95	62.94	62.96	62.94
(A) BiLSTM+GLFs	Bipolar	64.22	64.19	64.27	64.22
	Depression	62.58	62.59	62.60	62.59
	Stress	62.80	62.10	63.17	62.43
	ADHD	58.80	58.57	58.98	58.78
	Anxiety	64.81	64.81	64.81	64.80
(B) BiLSTM+LBFs	Bipolar	63.53	63.52	63.55	63.53
	Depression	62.21	62.20	62.22	62.21
	Stress	69.23	68.92	69.50	68.98
	ADHD	58.85	58.45	59.13	58.83
	Anxiety	62.80	62.52	63.21	62.81
(C) BiLSTM+GLFs+LBFs	Bipolar	62.23	61.72	62.81	62.25
	Depression	62.92	62.89	62.95	62.92
	Stress	71.03	70.78	71.10	70.83
	ADHD	64.28	64.72	64.68	64.65
	Anxiety	71.50	70.50	70.47	70.52
(D) Mental RoBERTa	Bipolar	71.55	71.83	72.04	71.62
	Depression	71.34	70.08	70.20	69.96
	Stress	82.22	81.62	81.65	81.59

Table 3 presents the results of the SP-LIME feature ablation experiments performed on the general linguistic features, whereas Table 4 presents the results of the same experiment on the lexicon-based features. The following ranking of GLFs for a given condition based on normalized feature importance scores are observed: The ADHD condition is best predicted by the group of stylistic features. The second most predictive group of features for this condition is that of lexical richness, followed by cohesion, morphosyntax and readability. The similar scores across the five feature groups suggest that they have comparable levels of importance in predicting the ADHD condition. The strongest predictor of anxiety condition is group cohesion, followed by morphosyntactic, lexical, and stylistic features, while readability features show the lowest predictive power. The group of lexical features is the strongest predictor of bipolar disorder condition, followed by cohesion, stylistic, morphosyntactic, and readability groups, in that order. When predicting depression, the most significant features are cohesive ones, followed by morphosyntactic, stylistic, and lexical features, whereas readability has a relatively minor impact.

**Table 3:** Normalized SP-LIME feature ablation results for General Linguistic Features (GLFs) in %.

**MHC**	**ADHD**	**Anxiety**	**Bipolar**	**Depression**	**Stress**
Cohesion	19.66 (4)	22.26 (1)	19.90 (2)	24.13 (1)	23.25 (2)
Lexical	20.51 (2)	21.08 (3)	22.97 (1)	20.60 (4)	13.12 (5)
Morphosyntatic	19.40 (3)	21.98 (2)	18.86 (4)	22.97 (2)	16.92 (3)
Stylistic	21.30 (1)	20.42 (4)	19.50 (3)	22.09 (3)	31.76 (1)
Readability	19.13 (5)	14.27 (5)	18.78 (5)	10.21 (5)	14.96 (4)

**Table 4:** Normalized SP-LIME feature ablation results for Lexicon-Based Features (LBFs) in %.

**MHC**	**ADHD**	**Anxiety**	**Bipolar**	**Depression**	**Stress**
ANEW	21.08 (1)	18.75 (1)	19.32 (1)	18.35 (1)	17.84 (3)
ANEW-Emo	17.01 (2)	10.23 (6)	11.66 (6)	8.24 (6)	22.86 (1)
EmoLex	13.35 (4)	16.27 (2)	15.36 (4)	17.13 (2)	9.68 (7)
GALC	13.43 (3)	15.91 (4)	16.04 (2)	16.76 (3)	10.06 (5)
General Inquirer	12.42 (6)	14.10 (5)	13.87 (3)	15.43 (5)	9.71 (6)
LIWC	10.13 (7)	8.62 (7)	9.48 (7)	8.05 (7)	11.88 (4)
SenticNet	12.58 (5)	16.11 (3)	14.27 (5)	16.03 (4)	17.97 (2)

Overall, in predicting five mental health conditions, the group of stylistic features had the strongest predictive power, with a total normalized importance score (Total I-score) of 115.07. Cohesion features were the second most influential group, with a Total I-score of 109.2, followed by morphosyntactic (Total I = 100.13) and lexical feature groups (I = 98.28). However, the importance of the readability feature group was relatively low, with a Total I-score of 98.28.

The normalized feature importance scores indicate the following ranking of LBFs for a given condition: When predicting the ADHD condition, the features from the ANEW lexicon are the strongest predictors, followed by features from ANEW-Emo, GALC, EmoLex, SenticNet, GI, and LIWC. In predicting the anxiety condition, the ANEW and EmoLex features are the primary predictors, followed by SenticNet, GALC, GI, and ANEW-Emo. The group of LIWC features shows the lowest predictive power. The best predictors for bipolar disorder are ANEW, GALC, and EmoLex features, followed by SenticNet, GI, ANEW-Emo, and LIWC. For depression, ANEW features are the strongest predictors, followed by EmoLex, GALC, SenticNet, and GI, while ANEW-Emo and LIWC play a minor role. In the case of psychological stress, the most effective predictors are ANEW-Emo, SenticNet, and ANEW, followed by LIWC, GALC, GI, and EmoLex.

In predicting mental health conditions, ANEW features are the strongest predictors overall, with a total importance score (Total I-score) of 95.34. SenticNet features are the second strongest predictors, with a Total I-score of 76.96, followed by GALC (Total I = 72.2), EmoLex (Total I = 71.79), ANEW-Emo (Total I = 70), and the GI feature group (Total I = 65.53). The LIWC feature group has the lowest importance overall, with a Total I-score of 48.1.

The results of the multi-task fusion models for each of the five mental health conditions are presented in Table 5, including the performance of each subtype of information infusion (emotion, personality, and both emotion and personality). As Table 5 shows, the best performing predictive model for the ADHD condition is with the emotion-infused model, achieving classification accuracy of 68.02%. In contrast, personality-infused models have the highest classification accuracy for the anxiety and bipolar conditions, achieving 73.40% and 73.23%, respectively. The full infusion models, incorporating both emotion and personality information, achieve the highest classification accuracies for depression and stress conditions, with 71.42% and 81.96%, respectively. Infusing personality and/or emotion information improves the average classification accuracy by 1.39% over the fine-tuned Mental RoBERTa baseline model, across all mental health conditions. The inclusion of personality information results in the greatest improvement for anxiety and bipolar disorder detection, with classification accuracy increases of +1.9% and +1.4%, respectively. ADHD detection sees the greatest improvement from the inclusion of emotion information, with a 3.74% increase in classification accuracy. For depression, infusing both personality and emotion information slightly improves classification accuracy by 0.08%.

**Table 5:** Performance comparison between type 3 multi-task fusion models.

**Model**	**MHC**	**Accuracy**	**F1-score**	**Precision**	**Recall**
	ADHD	68.02	67.12	67.77	66.58
	Anxiety	72.32	72.23	71.01	73.49
Emotion-Infused Model	Bipolar	71.49	70.10	69.75	70.45
	Depression	70.18	70.10	70.19	70.01
	Stress	81.12	81.05	81.23	81.00
	ADHD	67.99	67.65	67.56	67.74
	Anxiety	73.40	72.56	72.51	72.61
Personality-Infused Model	Bipolar	73.23	72.01	71.96	72.06
	Depression	68.33	67.13	66.80	67.46
	Stress	81.54	81.35	82.16	81.30
	ADHD	65.35	64.02	62.88	65.20
Emotion-Personality-Infused Model	Anxiety	72.36	72.88	72.63	73.13
	Bipolar	72.14	72.14	71.73	72.55
	Depression	71.42	71.46	71.46	71.46
	Stress	81.96	82.15	82.00	81.88

As previously stated in Section 1, our true goal in this work is to analyze the explainability of these models, building on the work of Turcan et al. (47). We now proceed to reporting on the two distinct analyses conducted to probe our trained models and determine what information they learn to use: (a) correlational analyses between the predictions of the primary task (MHC detection) and the auxiliary tasks (emotion detection and personality recognition), and (b) LIWC-based analyses designed to categorize the types of words utilized by Mental RoBERTa. Table 6 summarizes the results of correlations of the gold labels of the primary tasks (MHC detection) and the silver labels of the auxiliary task (emotion detection). The numbers reported represent the Pearson correlation coefficients that were computed on the test set. Our findings indicate that our emotion-infused multi-task models have learned to establish small to large correlations, with Pearson correlation coefficients ranging from 0.13 to 0.97, between the mental health labels and the emotion labels. Specifically, we found that the model has learned that the prediction of depression is strongly negatively correlated with anger (r = -0.47) and weakly positively correlated with sadness (r = 0.24) and fear (r = 0.22). Similarly, predictions of anxiety are negatively related to anger (r = -0.36), but positively related to the emotions of fear (r = 0.28), surprise (r = 0.27), joy (r = 0.21), and sadness (r = 0.18). On the other hand, for bipolar and ADHD conditions, the model has learned that each of them is associated with only a single emotion. In the case of bipolar disorder, positive cases are associated with the emotion of surprise (r = 0.53), while for ADHD, positive cases are associated with the presence of disgust (r = 0.38). The model has learned that stress is strongly negatively correlated with joy (r = -0.97) and moderately positively correlated with sadness (r=0.35).

**Table 6:** Correlations of the gold labels of the primary task and the silver labels of the auxiliary task in the emotion multi-task fusion model.

	**Anger**	**Disgust**	**Fear**	**Joy**	**Sadness**	**Surprise**
ADHD	−0.02	−0.13	−0.04	0.00	0.06	−0.01
Anxiety	−0.31	0.03	0.20	0.48	0.51	0.29
Bipolar	−0.06	0.01	0.00	0.06	−0.06	0.40
Depression	−0.53	−0.03	0.13	0.00	0.18	−0.02
Stress	0.04	0.00	0.03	−0.97	0.35	0.00

Table 7 presents a summary of the correlations between the gold labels of the primary tasks (MHC detection) and the silver labels of the auxiliary task (personality recognition). The results reveal that our personality-infused models have effectively learned to establish significant correlations, with Pearson correlation coefficients ranging from 0.11 to 0.93. The model has identified moderate positive associations between the prediction of ADHD condition and the Judging dimension (r = 0.30), weak positive associations with the Extraversion dimension (r = 0.11), and moderate negative associations with the Thinking dimension (r = -0.39). The model has learned that the prediction of anxiety is moderately positively associated with the Intuition dimension (r = 0.32), weakly positively associated with the Judging dimension (r = 0.13), and strongly negatively associated with the Thinking dimension (r = -0.73). The model has identified moderate positive associations between the prediction of depression and the Judging dimension (r = 0.22), and strong negative associations with the Thinking dimension (r = -0.93). The model has not learned any significant associations between stress prediction and the four personality dimensions.

**Table 7:** Correlations of the gold labels of the primary task and the silver labels of the auxiliary task in the personality multi-task fusion model.

	**Extraversion**	**Intuition**	**Thinking**	**Judging**
	**(vs Introversion)**	**(vs Sensing)**	**(vs Feeling)**	**(vs Perceiving)**
ADHD	0.11	0.02	−0.39	0.30
Anxiety	0.00	0.32	−0.78	0.13
Bipolar	0.12	0.08	−0.93	0.47
Depression	0.00	0.00	−0.93	0.22
Stress	0.00	0.00	−0.07	0.00

Table 8 shows the results of the LIWC-based analyses of how often our Type 2 (Mental RoBERTa) and Type 3 (Multi-Task Fusion) models rely on words from specific LIWC categories to make their decisions using the AGRAD method. The results obtained with the LIME method can be found in Supplementary Table S4 in the Supplementary material. We decided to focus on the AGRAD method as it provided more pronounced differences between the basic Mental RoBERTa models and the information-infused models compared to the results of the LIME method.

**Table 8:** Comparison of how often our models rely on words from several LIWC categories to make their decisions, according to AGRAD. These numbers represent the percentage of LIWC words each model selected in the top ten AGRAD explanations for the entire test set.

**Category**	**Model type**	**Bipolar**	**Depression**	**ADHD**	**Anxiety**	**Stress**
**Cognitive Processes**	**MentalRoBERTa**	**13.54**	**12.86**	**8.58**	**15.00**	**17.62**
Social Processes	MentalRoBERTa	5.63	5.49	8.45	4.83	5.11
Affective Processes	MentalRoBERTa	4.70	5.00	4.19	5.02	4.55
Cognitive Processes	+Emotion	13.21	14.26	12.98	14.67	16.06
Social	+Emotion	6.31	7.75	5.80	7.80	5.75
Affective Processes	+Emotion	4.60	4.32	4.14	4.33	4.36
Cognitive Processes	+Personality	11.52	11.27	12.52	14.20	11.22
Social Processes	+Personality	7.53	6.81	6.78	5.03	9.53
Affective Processes	+Personality	4.42	4.01	4.31	4.21	4.74
Cognitive Processes	+Emotion+Personality	15.6	14.75	11.86	13.95	14.13
Social Processes	+Emotion+Personality	5.80	5.04	6.36	8.46	6.31
Affective Processes	+Emotion+Personality	5.51	4.32	3.76	3.75	4.74

Zooming in on the basic MentalRoberta models across all five mental health conditions, we observe that the largest proportion of attended words come from “Cognitive Processes”, which covers 13.5% of the words driving its predictions. The words from the categories of “Social Processes” and “Affective Processes” account for 5.9% and 4.7% of those words, respectively. The general rank order of the relative coverage of these three LIWC categories is retained in the three multi-task fusion models. However, there are slight variations in the percentage of attended words among the three LIWC categories when comparing the basic RoBERTa model to the three multi-task fusion models. Notably, the models infused with emotions demonstrate a reduced reliance on words from the “Affective Processes” category across MHCs. On average, there is a decrease of 0.34% in the usage of such words compared to the RoBERTa baseline model. Instead, the emotion-infused models demonstrate a greater reliance on words that are associated with “Social Processes”. In particular, these models exhibit an increase in the use of words from this category when predicting four of the MHCs (ADHD, anxiety, depression, and stress), with the most noticeable difference being observed in predicting depression (an increase of +2.97%) and anxiety (an increase of +2.26%). Additionally, the prediction of bipolar disorder displays a notable increase in reliance on words related to cognitive processes, with an increase of 4.4%. Likewise, the personality-infused models exhibit an increased dependence on words related to “Social Processes” for all MHCs, except bipolar disorder, with the most significant difference (+4.4%) observed in the prediction of stress. In contrast to the emotion-infused models, the personality-infused models display slight increases in the usage of words from the “Affective Processes” category in the prediction of stress (+0.12%) and bipolar disorder (+0.19%). The models that incorporate both emotional and personality-related information exhibit distinct differences in their use of words compared to the other two multi-task fusion models. These differences include a higher emphasis on “Social Processes” for identifying depression (with a 0.66% increase compared to the emotion-infused model and a 3.42% increase compared to the personality-infused model) and a moderate decrease in the use of words associated with “Cognitive Processes” for predicting stress (with a 0.56% increase compared to the emotion-infused model and a 3.22% decrease compared to the personality-infused model).

## 7 Discussion

The overarching objective of our work was to improve the explainability and interpretability of the state-of-art literature on AI-assisted detection of mental disorders through the application of natural language processing and machine learning. In Section 1, we addressed three key research questions that were driving this work.

Our first research question (**RQ1**) was concerned with systematic comparisons between more interpretable mental health detection models and the state-of-the-art black box pre-trained, transformer-based language model to weigh the trade-off between interpretability and performance. To this end, we constructed and evaluated three Type 1 models, which were Bidirectional LSTM models trained on sequential information. Specifically, we used in-text distributions of two groups of interpretable features (GLFs and LBFs), as well as their combination. We then compared the performance of these models against a MentalRoBERTa baseline model. The F1-score difference between the more interpretable models and the baseline model varied across the five mental health conditions. The smallest difference was observed for anxiety, with a score of -5.69%, followed by ADHD (-6.15%), depression (-7.19%), bipolar disorder (-7.64%), and the largest difference was found for stress (-10.84%). The results of our experiments indicate that the average F1-score difference between the more interpretable models and the baseline model was 7.51% across the five mental health conditions. There are two potential reasons for why stress has a relatively higher differences compared to the other four mental health conditions. First, due to the subjectivity of stress and the different contexts where this concept is used, a universally recognized definition for stress is still lacking ((70)^4^). Second, the comparatively large difference in the case of stress must be evaluated in light of the methodological difference underlying the construction of the data sets used for ADHD, anxiety, bipolar and depression detection (SMHD) and those used for the stress detection (Dreaddit). As described in Section 3, in the SMHD dataset, positive labels were assigned based on self-disclosure of a diagnosis through the identification of self-reported diagnosis statements, such as “I was diagnosed with depression”. On the other hand, for the Dreaddit dataset, positive annotations were obtained through human annotators. Additionally, a critical distinction between the two datasets is that in SMHD to prevent easy inference of target labels from the presence of words and phrases indicative of MHC in posts, all posts made in subreddits pertaining to mental health or with keywords related to mental illness were excluded from the data of diagnosed users. The superior performance of the MentalRoBERTa model in detecting stress may be attributed to its ability to detect words and phrases related to stress. This is consistent with our findings presented in the previous section, where we observed that stress exhibited more distinctive linguistic signals for certain stylistic ngram features within the GLFs group and across a broad spectrum of sub-groups in the LBFs group, as compared to the other four mental health conditions. The presence of such strong content-related signals in language usage may be inevitable when relying on human assessment to establish ground truth for positive annotations.

Another finding related to our RQ1 is that using only GLFs for BiLSTM training was most effective for detecting bipolar disorder, while using LBFs was most effective for detecting anxiety. For the remaining three conditions (ADHD, depression, and stress), the best performing BiLSTM models included both GLFs and LBFs groups. This suggests that incorporating various types of interpretable features or their combinations should be considered when developing more transparent models for detecting specific mental health conditions. The choice of interpretable features to include in such models should be tailored to the specific mental health condition in question in order to build the most efficient and minimal adequate model possible.

Although the MentalRoBERTa achieved the highest accuracy in detecting mental health conditions, its lack of interpretability limits its usefulness in understanding which features contribute to its performance. Thus, in our second research question (**RQ2**), we aimed to determine which categories of features within the two groups (GLFs and LBFs) are the most predictive models in detecting mental health conditions. The results of our feature ablation experiments on GLFs indicated that distinct subgroups of features ranked differently in terms of their predictive power. Specifically, we found that stylistic features were the most predictive for detecting ADHD and psychological stress, while cohesion features were the most predictive for anxiety and depression. Interestingly, for bipolar disorder, lexical features were ranked as the most predictive. The morpho-syntactic feature group exhibited a moderate level of predictive power, occupying the 2nd to 4th rank in importance across all five mental health conditions. With the exception of the stress condition, the readability subgroup ranked last in terms of predictive power, indicating its relatively subsidiary role in the detection of mental illness. Correlational analyses of importance scores revealed that bipolar disorder, depression, and anxiety shared more similarities in terms of feature importance patterns, while they were markedly dissimilar from stress and ADHD. The observed pattern of results aligns with the existing body of evidence suggesting that comorbidity between depressive and anxiety disorders is prevalent (71). This pattern is also in agreement with clinical literature, which suggests that adults with ADHD may experience heightened susceptibility to daily life stressors (72).

Our experiments on LBFs indicated that ANEW (54) was the most predictive lexicon for four mental health conditions (ADHD, anxiety, bipolar, and depression), while ANEW-Emo (55), an extension of ANEW, was the most predictive for the stress condition. SenticNet (60), GALC (73), and EmoLex (74) had intermediate levels of predictive power across conditions. In contrast, the General Inquirer (56) and LIWC (75) lexicons were the least predictive feature groups. These findings suggest that relying solely on generic lexicons, such as LIWC and GI, may be less informative compared to using specialized emotion, sentiment, and affective lexicons for modeling and detecting mental health conditions. The observed higher feature importance ranking of the ANEW, ANEW-Emo, SenticNet, and EmoLex lexicons in contrast to the General Inquirer and LIWC lexicons may be explained by the fact that the former provide continuous scales to measure affective dimensions of words, whereas the latter two rely on measures that count the frequency and presence of words associated with specific categories.

For the third research question (**RQ3**), our focus was on constructing and assessing mental health classification models that incorporate information from multiple tasks, as well as examining the contribution of specific words to these models. The approach of infusing additional information resulted in slight enhancements in classification performance across all conditions, except for stress, with an average F1-score increase of 1.39%. The infusion of personality information led to the most substantial improvements in anxiety and bipolar detection, while emotion-infusion was most effective for ADHD detection. The detection of depression and stress both benefited from the incorporation of information from the auxiliary tasks. The lack of improvement in stress prediction through information infusion, as previously reported in Turcan et al. (47), could be attributed to the utilization of a baseline model in our work. In Turcan et al. (47), the baseline model used was the vanilla BERT, which attained an F1 score of 78.88%, whereas the MentalRoBERTa model we fine-tuned for our work achieved an F1 score of 82.22%. Our results suggest that incorporating information about individual personality traits can enhance the prediction of mental disorders. This finding is consistent with previous research indicating that certain personality traits are associated with a higher risk of developing depressive disorders (76–78). These studies have demonstrated that depression is more likely to develop in individuals with a combination of high neuroticism, low extraversion, and conscientiousness, according to the five-factor model of personality (79, 80).

The second aspect of **RQ3** involved utilizing model-agnostic and model-specific attention-based explanation techniques to determine which types of words each information-infused model relied on for making predictions. Our multi-task fusion models have learned significant correlations between mental health conditions and emotions and personality traits. The analysis based on AGRAD aimed to shed light on the models' preference for words falling under specific LIWC categories. The basic MentalRoBERTa model allocated the largest proportion of attention to words related to “Cognitive Processes,” followed by “Social Processes” and “Affective Processes,” which aligns with previous research linking cognitive words to rumination, a known sign of mental health conditions and chronic psychological stress [see Turcan et al. (47)]. The multi-task fusion models generally maintained the same rank order of these categories but exhibited variations in the percentage of attended words compared to the basic model. The models that incorporated both emotional and personality-related information displayed distinct differences in their use of words, including a higher emphasis on “Social Processes” for identifying depression and a moderate decrease in the use of words associated with “Cognitive Processes” for predicting stress. Taken together, our findings suggest that the framework for model interpretation based on emotion-enhanced multi-task models proposed in Turcan et al. (47) can be applied to the detection of various mental health conditions beyond stress, including ADHD, anxiety, bipolar disorder, and depression. The incorporation of personality information can also offer new dimensions of interpretability for these models.

There are a few limitations of the current study. While social media posts data hold a number of benefits pointed out in the previous section, it is important to acknowledge that our study lacked access to the more comprehensive information available to mental health professionals during the diagnostic process. For instance, crucial sociodemographic factors were not incorporated, which could significantly impact the performance of classification models. Addressing these factors in future research has the potential to enhance the quality and accuracy of deep learning models in this domain. Additionally, it is crucial to note that our data collection process focused solely on the social media platform Reddit, driven by reasons outlined in Section 2. To establish the generalizability and robustness of our predictive models, further investigation into model performance across diverse social media sources is necessary. Furthermore, although our study primarily focused on binary classification models, we recognize the intricate nature of mental disorders, characterized by comorbidity, which results in symptom overlap and diagnostic intricacies. As a result, we underscore the significance of developing multi-class models adept at discerning between diverse disorders. By incorporating multi-class classification, we can provide more comprehensive and accurate insights into mental health conditions, enabling a better understanding of the intricate relationships between different disorders and facilitating more informed and targeted interventions to improve mental health outcomes. In addition, conducting a longitudinal analysis of users' language behavior patterns through time-series data can significantly advance the development of a personalized detection model for mental illness at the individual level. This approach offers a dynamic lens through which we can comprehend the complexities of mental disorders. As we look ahead, our aim is to transcend the limitations of static methodologies and embrace a more dynamic standpoint, one that considers the temporal evolution of symptoms. This entails a specific focus on the dynamic interplay between linguistic biomarkers and the nuanced landscape of emotions and sentiments, allowing us to glean deeper insights into the manifestations of mental health conditions.

## 8 Conclusion

In our work, we aimed to address the lack of systematic assessments of explainability methods in the domain of mental health detection. To the best of our knowledge, our work is the first to provide a comprehensive assessment across five prominent mental health conditions (MHCs) using Natural Language Processing and Machine Learning. Our overall objective was to advance state-of-the-art research on explainable AI (XAI) approaches to automatic mental health detection from language behavior leveraging textual data from social media.

In pursuit of this objective, we present a comprehensive array of XAI methodologies, leveraging their individual strengths while acknowledging their limitations. Our first approach centers around the construction of models utilizing Bidirectional Long Short-Term Memory (BiLSTM) networks. These networks are trained on an extensive collection of human-interpretable features, designed to encapsulate the multifaceted nature of language. We then conduct extensive feature ablation experiments to dissect the contribution of each feature. The second approach revolves around enhancing the interpretability of Pretrained Language Models (PLMs), which have often been criticized for their opacity or “black box” nature. The PLM utilized in our study is a domain-specific language model tailored for mental healthcare (MentalRoBERTa). We adopt a multi-task fusion learning framework. This setup amalgamates information from two pertinent domains emotion and personality thus enhancing the interpretability of the PLM. To illuminate the workings of these models, we employ two distinct explanation techniques: the local interpretable model-agnostic explanations (LIME) method and a model-specific self-explaining method (AGRAD). These techniques are utilized to discern the specific categories of words that the basic MentalRoBERTa and the information-infused models rely on when generating predictions.

While predictive models utilizing basic MentalRoBERTa outperform the BiLSTM models in terms of detection accuracy across all five MHCs, the interpretability of models built on large pre-trained language models is restricted to analyzing attention values associated with individual words, and it cannot disclose indicators situated at higher levels of language analyses, such as syntactic properties and cohesion-related features. Conversely, decision-making in automatic MHC detection can strongly benefit from the inclusion of machine learning models that are trained on human interpretable features pertaining to multiple levels of linguistic organization. They serve as digital biomarkers and provide a great basis for digital phenotyping, i.e. computational analysis and derivation of an individual's behavioral and psychological characteristics, enabling the identification of potential signs of mental illness. In alignment with a broad characterization of markers in digital healthcare (81), we conceive of linguistic markers as digitally acquired, computationally derived measures of human language production. To grasp the varying significance of these human-interpretable features, we conducted a series of ablation experiments, training and evaluating our machine learning models with each group of features excluded individually. This approach allowed us to delve into the relative influence of each feature group on the prediction of mental disorders, providing valuable insights. Our research establishes a foundational milestone, poised to catalyze future investigations into machine learning models for mental health. These forthcoming studies, enriched with an expansive array of human-interpretable features spanning the entire spectrum of language behavior, can draw from our work as a cornerstone. This will facilitate cross-data-set comparisons of linguistic markers, spanning diverse mental health conditions. The synthesis of insights garnered from such endeavors will culminate in a methodical compendium of digital language biomarkers. These markers, demonstrated to hold predictive efficacy across a spectrum of studies and contextual nuances, can empower mental health practitioners in the early identification and continuous monitoring of mental health disorders.

## Data availability statement

The original contributions presented in the study are included in the article/Supplementary material, further inquiries can be directed to the corresponding authors.

## Author contributions

EK: Conceptualization: Ideas; formulation or evolution of overarching research goals and aims. Funding acquisition: Acquisition of the financial support for the project leading to this publication. Project administration: Management and coordination responsibility for the research activity planning and execution. Supervision: Oversight and leadership responsibility for the research activity planning and execution, including mentorship external to the core team. Writing – original draft: Preparation, creation and/or presentation of the published work, specifically writing the initial draft. Writing – review & editing: Preparation, creation and/or presentation of the published work by those from the original research group, specifically critical review, commentary, or revision – including pre or post-publication stages. SZ: Data curation: Management activities to annotate (produce metadata), scrub data and maintain research data (including software code, where it is necessary for interpreting the data itself) for initial use and later re-use. EK, SZ, YQ, and DW: Methodology: Development or design of methodology; creation of models. SZ and DW: Formal analysis: Application of statistical, mathematical, computational, or other formal techniques to analyze or synthesize study data. SZ, YQ, and DW: Software Programming, software development; designing computer programs; implementation of the computer code and supporting algorithms; testing of existing code components. YQ and DW: Validation Verification, whether as a part of the activity or separate, of the overall replication/reproducibility of results/experiments and other research outputs. All authors contributed to the article and approved the submitted version.

## Footnotes

^1^ https://www.who.int/news-room/fact-sheets/detail/mental-disorders

^2^ https://www.un.org/sustainabledevelopment/

^3^ https://www.personalitycafe.com/

^4^ https://www.stress.org/daily-life
