# Supplementary material for: Toward explainable AI (XAI) for mental health detection based on language behavior
Source: Front Psychiatry. 2023 Dec 7;14:1219479. doi: 10.3389/fpsyt.2023.1219479 (PMC10748510; doi:10.3389/fpsyt.2023.1219479)
Supplement: Supplementary file 1 [file Data_Sheet_1.PDF]

Supplementary Material

Table S1: Overview of the 194 General Linguistic Features covered in the present work

Feature Category	Code	Description
MORPHO-SYNTACTIC		
TYPE 1: LENGTH OF PRODUCTION UNIT		
Mean length of clause (words)	MLC	# of words / # of clauses
Mean length of sentence (words)	MLS	# of words / # of sentences
Mean length of T-unit (words)	MLT	# of words / # of T-units
TYPE 2: SENTENCE COMPLEXITY		
Clauses per sentence	C/S	# of clauses / # of sentences
TYPE 3: SUBORDINATION		
Clauses per T-unit	C/T	# of clauses / # of T-units
Complex T-unit per T-unit	CompT/T	# of complex T-units / # of T-units
Dependent clauses per clause	DepC/C	# of dependent clauses / # of clauses
Dependent clauses per T-unit	DepC/T	# of dependent clauses / # of T-units
TYPE 4: COORDINATION		
Coordinate phrases per clause	CoordP/C	# of coordinate phrases / # of clauses
Coordinate phrases per T-unit	CoordP/T	# of coordinate phrases / # of T-units
T-units per sentence	T/S	# of T-units / # of sentences
TYPE 5: PARTICULAR STRUCTURES		
Complex nominals per clause	CompN/C	# of complex nominals / # of clauses
Complex nominals per T-unit	CompN/T	# of complex nominals / # of T-units
Verb phrases per T-unit	VP/T	# of verb phrases / # of T-units
Noun phrase pre-modification	NP.PreMod	# premodifying words in NP / # NPs
Noun phrase post-modification	NP.PostMod	# postmodifying words in NP / # NPs
TYPE 6: INFORMATION-THEORETIC MEASURES		
Kolmogorov Deflate	KolDef	#bytes after deflation / #bytes original
Morphological Kolmogorov Deflate	morKolDef	#bytes after character drop / #bytes original
Syntactic Kolmogorov Deflate	synKolDef	#bytes after word drop / #bytes original
LEXICAL		
TYPE 1: LEXICAL DIVERSITY		
Number of different words	NDW	# of word types
Corrected Number of different words	cNDW	# of word types
Type-Token Ratio (TTR)	TTR	# of word types / # of word tokens
Corrected TTR	cTTR	# of word types / squareroot (# of word tokens)

Continued on next page

Table S1 – continued from previous page

Feature group	Code	Description
Root TTR	rTTR	# of word types / squareroot (2 # of word tokens)
TYPE 2: LEXICAL SOPHISTICATION		
NAWL word ratio	NAWL	#words on NAWL / #words
AFL word ratio	AFL	#words on AFL / #words
ANC word ratio	ANC	#words on top 2000 of ANC / #words
BNC word ratio	BNC	#words on top 2000 of BNC / #words
NGSL word ratio	NGSL	#words on NGSL / #words
Average age of acquisition	AoA-mean	$\sum$mean AoA scores / #words in list
Maximum age of acquisition	AoA-max	max(mean AoA scores)
Mean length per word (characters)	MLWc	#characters / #words
Mean length per word (syllables)	MLWs	#syllables / #words
Nonstopword ratio	NonStop	#words not on NLTK stopword list / #words
TYPE 3: LEXICAL DENSITY		
Lexical density	LD	# of contents words / # of words
TYPE 4: WORD PREVALENCE		
Crowdsourcing-based	WordPrev	$\sum_w prevalence_w$ / #words
Corpus-based		#words in a category / #words
	AllAP	AP = author prevalence
	AllBP	BP = book prevalence
	AllCD	CD = contextual diversity
	AllSD	SD = semantic diversity
	AllSDAP	
	AllSDBP	
	AllWF	WF = word frequency
	FemaleAP	
	FemaleBP	Female = only female authors
	FemaleCD	
	FemaleSD	
	FemaleSDAP	
	FemaleSDBP	
	FemaleWF	
	MaleAP	Male = only male authors
	MaleBP	
	MaleCD	
	MaleSD	
	MaleSDAP	
	MaleSDBP	
	MaleWF	
	UKAP	UK = British English
	UKBP	
	UKCD	

Continued on next page

Table S1 – continued from previous page

Feature group	Code	Description
	UKSDAP	
	UKSDBP	
	UKWF	
	USAAP	USA = American English
	USABP	
	USACD	
	USASD	
	USASDAP	
	USASDBP	
	USAWF	
READABILITY		
Flesh-Kincaid Reading Ease	FKRE	$206.835 - 1.015MLS - 84.6MLWs$
Flesh-Kincaid Grade Level	FKGL	$0.39MLS - 11.8MLWs - 15.59$
Simple Measure of Gobbledygook	SMOG	$1.043\sqrt{\frac{30\#words_{syl\geq3}}{\#sentences}} + 3.129$
ColemanLiau	CL	$100(0.0588MLWc - \frac{0.296}{MLS}) - 15.8$
LIX index	LIX	$MLS + \frac{100\#words_{char>6}}{\#words}$
Automated Readability Index	ARI	$4.71MLWc - 0.5MLS - 21.43$
Dale-Chall	DS	$0.1579\frac{100\#hard\_words}{\#words} + 0.0496MLS$
Fry	FRY	x: $100MLWs$, y: MLS
Gunning Fox Index	GFI	$0.4(MLS + \frac{100\#words_{syl\geq3}}{\#words})$
Powers-Sumner-Kearl version of Dale and Chall	DSPSK	$0.1155(\frac{100\#hard\_words}{\#words}) + 0.596MLS + 3.2672$
FORCAST	FORCAST	$20 - \frac{150\#words_{syl=1}}{10\#words}$
Readability Index	RIX	$\frac{\#words_{syl\geq7}}{\#sentences}$
Spache	SPC	$0.121MLS + 0.82\frac{\#words_{notinspache}}{\#words} + 0.652$
COHESION		
TYPE 1: LEXICAL OVERLAP		
Overlap all lemmas	OL	# of all lemma types in current and next sentence / # of lemma types in current sentence
Overlap all lemmas (sentence normed)	NormedOL	# of all lemma types in current and next sentence / # of sentences in text - 1
Binary overlap all lemmas	BOL	# of sentences with any lemma overlap/# of sentences in text - 1
Two-sentence overlap all lemmas	2SOL	# of all lemma types in current and next two sentences / # of lemma types in current sentence
Two-sentence overlap all lemmas (sentence normed)	Normed2SOL	# of all lemma types in current and next two sentences / # of sentences in text - 2
Binary two-sentence overlap all lemmas	B2OL	# of sentences with any lemma overlap/# of sentences in text - 2

Continued on next page

Table S1 – continued from previous page

Feature group	Code	Description
Overlap content lemmas	OCL	# of content lemma types in current and next sentence / # of lemma types in current sentence
Overlap content lemmas (sentence normed)	NormedOCL	# of content lemma types in current and next sentence / # of sentences in text - 1
Binary overlap content lemmas	BOCL	# of sentences with any content lemma overlap/# of sentences in text - 1
Two-sentence overlap content lemmas	2OCL	# of content lemma types in current and next two sentences / # of lemma types in current sentence
Two-sentence overlap content lemmas (sentence normed)	Normed2OCL	# of content lemma types in current and next two sentences / # of sentences in text - 2
Binary two-sentence overlap content lemmas	BOCL	# of sentences with any content lemma overlap/# of sentences in text - 2
Overlap function lemmas	OFL	# of function lemma types in current and next sentence / # of lemma types in current sentence
Overlap function lemmas (sentence normed)	NormedOFL	# of function lemma types in current and next sentence / # of sentences in text - 1
Binary overlap function lemmas	BOFL	# of sentences with any function lemma overlap/# of sentences in text - 1
Two-sentence overlap function lemmas	2OFL	# of function lemma types in current and next two sentences / # of lemma types in current sentence
Two-sentence overlap function lemmas (sentence normed)	Normed2OFL	# of function lemma types in current and next two sentences / # of sentences in text - 2
Binary two-sentence overlap function lemmas	BOFL	# of sentences with any function lemma overlap/# of sentences in text - 2
Overlap noun lemmas	ONL	# of noun lemma types in current and next sentence / # of lemma types in current sentence
Overlap noun lemmas (sentence normed)	NormedONL	# of noun lemma types in current and next sentence / # of sentences in text - 1
Binary overlap noun lemmas	BONL	# of sentences with any noun lemma overlap/# of sentences in text - 1
Two-sentence overlap noun lemmas	2ONL	# of noun lemma types in current and next two sentences / # of lemma types in current sentence
Two-sentence overlap noun lemmas (sentence normed)	Normed2ONL	# of noun lemma types in current and next two sentences / # of sentences in text - 2
Binary two-sentence overlap noun lemmas	BONL	# of sentences with any noun lemma overlap/# of sentences in text - 2
Overlap verb lemmas	OVL	# of verb lemma types in current and next sentence / # of lemma types in current sentence
Overlap verb lemmas (sentence normed)	NormedOVL	# of verb lemma types in current and next sentence / # of sentences in text - 1
Binary overlap verb lemmas	BOVL	# of sentences with any verb lemma overlap/# of sentences in text - 1

Continued on next page

Table S1 – continued from previous page

Feature group	Code	Description
Two-sentence overlap verb lemmas	2OVL	# of verb lemma types in current and next two sentences / # of lemma types in current sentence
Two-sentence overlap verb lemmas (sentence normed)	Normed2OVL	# of verb lemma types in current and next two sentences / # of sentences in text - 2
Binary two-sentence overlap verb lemmas	BOVL	# of sentences with any verb lemma overlap/# of sentences in text - 2
Overlap adjective lemmas	OAdjL	# of adjective lemma types in current and next sentence / # of lemma types in current sentence
Overlap adjective lemmas (sentence normed)	NormedOAdjL	# of adjective lemma types in current and next sentence / # of sentences in text - 1
Binary overlap adjective lemmas	BOAdjL	# of sentences with any adjective lemma overlap/# of sentences in text - 1
Two-sentence overlap adjective lemmas	2OAdjL	# of adjective lemma types in current and next two sentences / # of lemma types in current sentence
Two-sentence overlap adjective lemmas (sentence normed)	Normed2OAdjL	# of adjective lemma types in current and next two sentences / # of sentences in text - 2
Binary two-sentence overlap adjective lemmas	BOAdjL	# of sentences with any adjective lemma overlap/# of sentences in text - 2
Overlap adverb lemmas	OAdvL	# of adverb lemma types in current and next sentence / # of lemma types in current sentence
Overlap adverb lemmas (sentence normed)	NormedOAdvL	# of adverb lemma types in current and next sentence / # of sentences in text - 1
Binary overlap adverb lemmas	BOAdvL	# of sentences with any adverb lemma overlap/# of sentences in text - 1
Two-sentence overlap adverb lemmas	2OAdvL	# of adverb lemma types in current and next two sentences / # of lemma types in current sentence
Two-sentence overlap adverb lemmas (sentence normed)	Normed2OAdvL	# of adverb lemma types in current and next two sentences / # of sentences in text - 2
Binary two-sentence overlap adverb lemmas	BOAdvL	# of sentences with any adverb lemma overlap/# of sentences in text - 2
Overlap pronoun lemmas	OPL	# of pronouns lemma types in current and next sentence / # of lemma types in current sentence
Overlap pronoun lemmas (sentence normed)	NormedOPL	# of pronouns lemma types in current and next sentence / # of sentences in text - 1
Binary overlap pronoun lemmas	BOPL	# of sentences with any pronouns lemma overlap/# of sentences in text - 1
Two-sentence overlap pronoun lemmas	2OPL	# of pronouns lemma types in current and next two sentences / # of lemma types in current sentence
Two-sentence overlap pronoun lemmas (sentence normed)	Normed2OPL	# of pronouns lemma types in current and next two sentences / # of sentences in text - 2

Continued on next page

Table S1 – continued from previous page

Feature group	Code	Description
Binary two-sentence overlap pronoun lemmas	BOPL	# of sentences with any pronouns lemma overlap/# of sentences in text - 2
Overlap noun and pronoun lemmas	ONPL	# of noun and pronoun lemma types in current and next sentence / # of lemma types in current sentence
Overlap noun and pronoun lemmas (sentence normed)	NormedONPL	# of noun and pronoun lemma types in current and next sentence / # of sentences in text - 1
Binary overlap noun and pronoun lemmas	BONPL	# of sentences with any noun and pronoun lemma overlap/# of sentences in text - 1
Two-sentence overlap noun and pronoun lemmas	2ONPL	# of noun and pronoun lemma types in current and next two sentences / # of lemma types in current sentence
Two-sentence overlap noun and pronoun lemmas (sentence normed)	Normed2ONPL	# of noun and pronoun lemma types in current and next two sentences / # of sentences in text - 2
Binary two-sentence overlap noun and pronoun lemmas	BONPL	# of sentences with any noun and pronoun lemma overlap/# of sentences in text - 2
TYPE 2: CONNECTIVES		
Addition	ADDW	# of addition words
All	CONJW	# of conjunctions
All.additive	ACONJ	# of additive connectives
All.causal	CCONN	# of causal connectives
All.demonstratives	DEMO	# of demonstratives
All.logical	LCONN	# of logical connectives
All.negative	NCONN	# of negative connectives
All.positive	PCONN	# of positive connectives
Basic	BCONN	# of basic connectives
Conjunctions	CONJ	# of conjunctions
Coordinating.conjuncts	CO-CONJ	# of coordinating conjuncts
Determiners	DET	# of determiners
Disjunctions	DISJ	# of disjunctions
Lexical.subordinators	lex.SUB	# of lexical items functioning as subordinators
Negative.logical	neg.LCONN	# of negative logical connectives
Opposition	OPPW	# of opposition words
Order	ORDW	# of order words
Positive.causal	pos.CCONN	# of positive causal connectives
Positive.intentional	pos.ICONN	# of positive intentional connectives
Positive.logical	pos.LCONN	# of positive logical connectives
Reason.and.purpose	RPW	# of reason and purpose words
Sentence.linking	SLW	# of sentence linking words
Temporal	TCONN	# of temporal connectives
STYLISTIC - REGISTER-BASED N-GRAM		

Continued on next page

Table S1 – continued from previous page

Feature group	Code	Description
Unigram frequency score academic	Unigram.acad	# of ngrams in list / sum word log(Freq)
Bigram frequency score academic	Bigram.acad	# of ngrams in list / sum word log(Freq)
Trigram frequency score academic	Trigram.acad	# of ngrams in list / sum word log(Freq)
Fourgram frequency score academic	Fourgram.acad	# of ngrams in list / sum word log(Freq)
Unigram frequency score blog	Unigram.blog	# of ngrams in list / sum word log(Freq)
Bigram frequency score blog	Bigram.blog	# of ngrams in list / sum word log(Freq)
Trigram frequency score blog	Trigram.blog	# of ngrams in list / sum word log(Freq)
Fourgram frequency score blog	Fourgram.blog	# of ngrams in list / sum word log(Freq)
Unigram frequency score fiction	Unigram.fic	# of ngrams in list / sum word log(Freq)
Bigram frequency score fiction	Bigram.fic	# of ngrams in list / sum word log(Freq)
Trigram frequency score fiction	Trigram.fic	# of ngrams in list / sum word log(Freq)
Fourgram frequency score fiction	Fourgram.fic	# of ngrams in list / sum word log(Freq)
Unigram frequency score mag	Unigram.mag	# of ngrams in list / sum word log(Freq)
Bigram frequency score mag	Bigram.mag	# of ngrams in list / sum word log(Freq)
Trigram frequency score mag	Trigram.mag	# of ngrams in list / sum word log(Freq)
Fourgram frequency score mag	Fourgram.mag	# of ngrams in list / sum word log(Freq)
Unigram frequency score news	Unigram.news	# of ngrams in list / sum word log(Freq)
Bigram frequency score news	Bigram.news	# of ngrams in list / sum word log(Freq)
Trigram frequency score news	Trigram.news	# of ngrams in list / sum word log(Freq)
Fourgram frequency score news	Fourgram.news	# of ngrams in list / sum word log(Freq)
Unigram frequency score spoken	Unigram.spok	# of ngrams in list / sum word log(Freq)
Bigram frequency score spoken	Bigram.spok	# of ngrams in list / sum word log(Freq)
Trigram frequency score spoken	Trigram.spok	# of ngrams in list / sum word log(Freq)
Fourgram frequency score spoken	Fourgram.spok	# of ngrams in list / sum word log(Freq)
Unigram frequency score tv movies	Unigram.tvm	# of ngrams in list / sum word log(Freq)
Bigram frequency score tv movies	Bigram.tvm	# of ngrams in list / sum word log(Freq)
Trigram frequency score tv movies	Trigram.tvm	# of ngrams in list / sum word log(Freq)
Fourgram frequency score tv movies	Fourgram.tvm	# of ngrams in list / sum word log(Freq)
Unigram frequency score web	Unigram.web	# of ngrams in list / sum word log(Freq)
Bigram frequency score web	Bigram.web	# of ngrams in list / sum word log(Freq)
Trigram frequency score web	Trigram.web	# of ngrams in list / sum word log(Freq)

Table S2: Pairwise comparisons of mean scores of General Language Features (GLFs) between each group of mental health condition and the respective control group. Numbers in the cells represent effect sizes (Cohen's d). Colors indicate magnitude of effect size: no color: $d < 0.1$, light-blue: $d > 0.1$, blue: > 0.2, darker blue: $d > 0.5$, dark blue: $d > 0.8$. Only features that are significant at $\alpha = 0.01$ for at least one MHC are included.

Feature	Group	Bipolar	Depression	ADHD	Anxiety	Stress
addition	cohesion	0.07	0.05	–	0.04	0.23
all	cohesion	0.07	0.05	–	0.06	0.16
all.additive	cohesion	0.07	0.05	–	0.05	0.21
all.causal	cohesion	0.11	0.11	0.08	0.12	0.24
all.logical	cohesion	0.06	–	0.06	0.05	–
all.positive	cohesion	0.06	0.07	–	0.07	0.2
basic	cohesion	0.12	0.09	0.04	0.09	0.3
conjunctions	cohesion	0.1	0.07	–	0.07	0.36
coordinating.conjuncts	cohesion	0.07	0.09	0.05	0.09	0.15
determiners	cohesion	-0.15	-0.16	-0.08	-0.18	-0.61
lexical.subordinators	cohesion	0.06	0.04	0.06	0.06	-0.12
order	cohesion	-0.05	-0.04	–	-0.05	–
positive.causal	cohesion	–	0.05	0.05	0.06	0.26
positive.intentional	cohesion	0.07	0.07	–	0.07	0.15
reason.and.purpose	cohesion	0.06	0.08	0.06	0.09	0.14
sentence.linking	cohesion	0.05	0.05	–	0.06	–
temporal	cohesion	-0.06	–	-0.05	–	-0.08
2.adj.sent	cohesion	0.05	–	–	–	–
2.adj.sent.div.seg	cohesion	–	–	–	–	-0.08
2.adv.sent	cohesion	0.16	0.16	0.1	0.16	0.32
2.adv.sent.div.seg	cohesion	–	0.05	–	–	0.25
2.all.sent	cohesion	0.32	0.29	0.25	0.3	0.55
2.all.sent.div.seg	cohesion	–	–	–	–	0.19
2.arg.sent	cohesion	0.27	0.25	0.2	0.27	0.5
2.arg.sent.div.seg	cohesion	–	–	–	–	0.22
2.cw.sent	cohesion	0.21	0.19	0.16	0.19	0.23
2.cw.sent.div.seg	cohesion	–	–	–	-0.05	–
2.fw.sent	cohesion	0.32	0.3	0.25	0.31	0.66
2.fw.sent.div.seg	cohesion	–	–	–	–	0.27
2.noun.sent	cohesion	–	-0.06	–	-0.07	-0.34
2.noun.sent.div.seg	cohesion	-0.14	-0.12	-0.07	-0.14	-0.34
2.prn.sent	cohesion	0.39	0.37	0.26	0.41	0.88
2.prn.sent.div.seg	cohesion	0.11	0.13	0.05	0.14	0.68
2.verb.sent	cohesion	0.3	0.27	0.21	0.29	0.52
2.verb.sent.div.seg	cohesion	–	0.05	–	–	0.3
adj.sent	cohesion	–	–	–	–	-0.09
adj.sent.div.seg	cohesion	-0.05	–	–	–	-0.16
adv.sent	cohesion	0.09	0.11	0.06	0.11	0.24

Feature	Group	Bipolar	Depression	ADHD	Anxiety	Stress
all.sent	cohesion	0.19	0.2	0.18	0.21	0.43
all.sent.div.seg	cohesion	-0.15	-0.08	-0.08	-0.1	-0.2
arg.sent	cohesion	0.19	0.2	0.15	0.22	0.51
arg.sent.div.seg	cohesion	-0.13	-0.04	-0.07	-0.05	-0.07
bi.2.adv.sent	cohesion	0.1	0.12	0.07	0.1	0.35
bi.2.all.sent	cohesion	0.23	0.19	0.16	0.19	0.55
bi.2.arg.sent	cohesion	0.22	0.2	0.17	0.21	0.71
bi.2.cw.sent	cohesion	0.19	0.15	0.13	0.14	0.4
bi.2.fw.sent	cohesion	0.22	0.18	0.16	0.19	0.61
bi.2.noun.sent	cohesion	-0.08	-0.07	–	-0.09	-0.31
bi.2.prn.sent	cohesion	0.3	0.29	0.2	0.31	0.95
bi.2.verb.sent	cohesion	0.19	0.18	0.14	0.18	0.5
bi.adj.sent	cohesion	–	–	–	–	-0.13
bi.adv.sent	cohesion	–	0.07	–	0.06	0.18
bi.all.sent	cohesion	0.09	0.12	0.1	0.12	0.3
bi.arg.sent	cohesion	0.12	0.17	0.12	0.18	0.51
bi.cw.sent	cohesion	–	0.05	0.07	0.05	0.07
bi.fw.sent	cohesion	0.08	0.12	0.1	0.12	0.38
bi.noun.sent	cohesion	-0.18	-0.12	-0.06	-0.16	-0.49
bi.prn.sent	cohesion	0.23	0.26	0.17	0.28	0.77
bi.verb.sent	cohesion	0.08	0.11	0.1	0.11	0.22
cw.sent	cohesion	0.09	0.09	0.1	0.1	0.09
cw.sent.div.seg	cohesion	-0.16	-0.09	-0.08	-0.11	-0.27
fw.sent	cohesion	0.21	0.22	0.19	0.23	0.56
fw.sent.div.seg	cohesion	-0.13	-0.06	-0.07	-0.08	-0.12
noun.sent	cohesion	-0.13	-0.12	–	-0.12	-0.39
noun.sent.div.seg	cohesion	-0.2	-0.13	-0.1	-0.15	-0.43
prn.sent	cohesion	0.33	0.33	0.22	0.37	0.88
prn.sent.div.seg	cohesion	–	0.07	–	0.07	0.3
verb.sent	cohesion	0.2	0.19	0.16	0.21	0.38
verb.sent.div.seg	cohesion	-0.09	–	-0.04	–	-0.08
1gram.acad	stylistic	–	–	–	–	0.38
1gram.blog	stylistic	-0.12	-0.06	-0.05	-0.11	-0.12
1gram.fic	stylistic	-0.1	-0.04	–	-0.09	-0.08
1gram.mag	stylistic	-0.11	-0.06	–	-0.1	-0.12
1gram.news	stylistic	-0.12	-0.06	-0.05	-0.11	-0.12
1gram.spok	stylistic	-0.1	-0.04	–	-0.09	-0.08
1gram.tvm	stylistic	-0.1	-0.04	–	-0.09	-0.08
1gram.web	stylistic	-0.12	-0.06	-0.05	-0.11	-0.13
2gram.blog	stylistic	–	–	–	–	0.08
2gram.fic	stylistic	–	0.08	0.05	0.05	0.21
2gram.mag	stylistic	–	–	–	–	0.08
2gram.news	stylistic	–	–	–	–	0.09
2gram.spok	stylistic	–	0.05	–	–	0.15

Feature	Group	Bipolar	Depression	ADHD	Anxiety	Stress
2gram.tvm	stylistic	–	0.07	–	–	0.2
2gram.web	stylistic	–	–	–	–	0.09
3gram.acad	stylistic	–	0.04	0.06	–	0.07
3gram.blog	stylistic	–	0.08	0.06	0.06	0.19
3gram.fic	stylistic	0.07	0.12	0.06	0.09	0.37
3gram.mag	stylistic	–	0.07	0.06	–	0.16
3gram.news	stylistic	–	0.06	0.04	–	0.2
3gram.spok	stylistic	–	0.08	0.06	0.06	0.23
3gram.tvm	stylistic	0.06	0.11	0.04	0.09	0.33
3gram.web	stylistic	–	0.09	0.06	0.06	0.22
4gram.blog	stylistic	–	0.06	0.05	0.06	0.15
4gram.fic	stylistic	0.05	0.1	0.05	0.09	0.33
4gram.mag	stylistic	–	–	0.05	–	0.13
4gram.news	stylistic	–	0.05	0.04	–	0.17
4gram.spok	stylistic	–	0.05	0.05	–	0.19
4gram.tvm	stylistic	–	0.07	–	0.06	0.27
4gram.web	stylistic	–	0.07	0.05	0.06	0.19
AFL	lexical	–	–	–	–	-0.12
ANC	lexical	-0.32	-0.32	-0.25	-0.34	-0.72
AoA.max	lexical	-0.12	-0.16	–	-0.18	-0.42
AoA.mean	lexical	-0.11	-0.16	–	-0.17	-0.43
BNC	lexical	-0.21	-0.22	-0.17	-0.22	-0.44
CNDW	lexical	0.08	–	–	0.06	-0.18
cTTR	lexical	-0.2	-0.16	-0.1	-0.19	-0.35
LexDens	lexical	-0.18	-0.22	-0.15	-0.24	-0.64
MLWc	lexical	-0.12	-0.15	-0.05	-0.16	-0.57
MLWs	lexical	–	-0.04	0.05	–	-0.27
NAWL	lexical	–	–	0.06	–	-0.17
NDW	lexical	-0.2	-0.14	-0.1	-0.19	-0.29
NGSL	lexical	-0.28	-0.26	-0.2	-0.28	-0.55
NonStopWordsRate	lexical	-0.24	-0.24	-0.17	-0.26	-0.71
Prev.AllAP	lexical	0.31	0.25	0.2	0.28	0.43
Prev.AllBP	lexical	0.31	0.26	0.2	0.29	0.47
Prev.AllCD	lexical	0.32	0.28	0.2	0.3	0.54
Prev.AllSD	lexical	0.32	0.27	0.2	0.3	0.48
Prev.AllSDAP	lexical	0.31	0.26	0.2	0.29	0.47
Prev.AllSDBP	lexical	0.32	0.28	0.21	0.3	0.51
Prev.AllWF	lexical	0.31	0.27	0.2	0.3	0.54
Prev.FemaleAP	lexical	0.31	0.26	0.2	0.29	0.45
Prev.FemaleBP	lexical	0.32	0.27	0.21	0.3	0.49
Prev.FemaleCD	lexical	0.32	0.28	0.21	0.31	0.56
Prev.FemaleSD	lexical	0.32	0.27	0.21	0.3	0.49
Prev.FemaleSDAP	lexical	0.32	0.27	0.21	0.3	0.5
Prev.FemaleSDBP	lexical	0.33	0.29	0.21	0.32	0.55

Feature	Group	Bipolar	Depression	ADHD	Anxiety	Stress
Prev.FemaleWF	lexical	0.32	0.28	0.2	0.31	0.57
Prev.MaleAP	lexical	0.3	0.25	0.19	0.27	0.42
Prev.MaleBP	lexical	0.31	0.26	0.2	0.28	0.46
Prev.MaleCD	lexical	0.32	0.28	0.2	0.3	0.54
Prev.MaleSD	lexical	0.32	0.27	0.2	0.3	0.48
Prev.MaleSDAP	lexical	0.31	0.26	0.2	0.28	0.47
Prev.MaleSDBP	lexical	0.31	0.27	0.2	0.29	0.51
Prev.MaleWF	lexical	0.31	0.27	0.2	0.29	0.53
Prev.UKAP	lexical	0.31	0.25	0.2	0.28	0.45
Prev.UKBP	lexical	0.32	0.27	0.2	0.29	0.49
Prev.UKCD	lexical	0.32	0.28	0.2	0.31	0.56
Prev.UKSD	lexical	0.32	0.28	0.21	0.3	0.52
Prev.UKSDAP	lexical	0.32	0.27	0.2	0.29	0.51
Prev.UKSDBP	lexical	0.32	0.28	0.21	0.31	0.55
Prev.UKWF	lexical	0.31	0.28	0.2	0.3	0.55
Prev.USAAP	lexical	0.31	0.25	0.2	0.28	0.43
Prev.USABP	lexical	0.31	0.26	0.2	0.29	0.47
Prev.USACD	lexical	0.32	0.28	0.2	0.31	0.55
Prev.USASD	lexical	0.32	0.27	0.21	0.3	0.49
Prev.USASDAP	lexical	0.31	0.27	0.2	0.29	0.48
Prev.USASDBP	lexical	0.32	0.28	0.21	0.3	0.52
Prev.USAWF	lexical	0.31	0.28	0.2	0.3	0.55
rTTR	lexical	-0.21	-0.16	-0.1	-0.19	-0.35
TTR	lexical	0.08	–	–	0.06	-0.18
WordPrev	lexical	0.34	0.27	0.23	0.3	0.46
Base.Kolmogorov	morphosyntactic	0.11	0.09	0.06	0.1	0.23
CompNompC	morphosyntactic	-0.18	-0.21	-0.11	-0.24	-0.47
CompNompT	morphosyntactic	-0.17	-0.14	-0.08	-0.19	-0.49
CompTpT	morphosyntactic	–	–	–	–	-0.13
CoordPpC	morphosyntactic	–	-0.07	–	-0.08	–
CoordPpT	morphosyntactic	–	-0.07	-0.04	-0.09	–
CpS	morphosyntactic	-0.06	–	–	–	–
CpT	morphosyntactic	-0.07	–	–	-0.05	-0.08
DCpC	morphosyntactic	-0.11	-0.07	–	-0.07	-0.28
DCpT	morphosyntactic	-0.09	-0.04	–	-0.06	-0.2
KolDef	morphosyntactic	0.1	0.09	0.05	0.09	0.23
MLC	morphosyntactic	-0.16	-0.17	-0.1	-0.19	-0.21
MLS	morphosyntactic	-0.18	-0.12	-0.1	-0.17	-0.23
MLT	morphosyntactic	-0.16	-0.12	-0.08	-0.15	-0.29
MorKol	morphosyntactic	0.1	0.09	0.05	0.09	0.22
NP.PostMod	morphosyntactic	-0.12	-0.11	-0.1	-0.15	-0.17
NP.PreMod	morphosyntactic	-0.15	-0.14	-0.08	-0.12	-0.32
TpS	morphosyntactic	0.05	0.07	–	0.08	0.24
VPpT	morphosyntactic	-0.09	–	–	-0.07	-0.14

Feature	Group	Bipolar	Depression	ADHD	Anxiety	Stress
ARI	readability	-0.19	-0.14	-0.09	-0.2	-0.33
ColemanLiau	readability	-0.17	-0.11	-0.09	-0.17	-0.21
DaleChall	readability	-0.2	-0.18	-0.13	-0.18	-0.23
DaleChallPSK	readability	-0.24	-0.19	-0.15	-0.22	-0.29
FKGL	readability	-0.16	-0.11	-0.07	-0.17	-0.27
FKRE	readability	0.14	0.11	0.04	0.15	0.32
FORCAST	readability	–	-0.08	–	-0.08	-0.26
Fry.x	readability	–	-0.04	0.05	–	-0.27
GunningFog	readability	-0.17	-0.11	-0.09	-0.17	-0.21
Lix	readability	-0.17	-0.12	-0.08	-0.18	-0.27
Rix	readability	-0.16	-0.1	-0.09	-0.15	-0.17
SMOG	readability	–	–	0.09	–	-0.14
Spache	readability	-0.17	-0.11	-0.09	-0.17	-0.21

Table S3: Differences in Lexicon-Based Features (LBFs) between affected and control users. Numbers in the cells represent effect sizes. The colors indicate four levels of effect size measured via Cohen's d: small: A commonly used interpretation is to refer to effect sizes as small ($d = 0.2$), medium ($d = 0.5$), and large ($d = 0.8$).

Feature	Dictionary	Bipolar	Depression	ADHD	Anxiety	Stress
ANEW.pleasure	ANEW	0.13	0.06	–	0.06	-0.06
ANEW.arousal	ANEW	0.13	0.08	–	0.07	0.19
ANEW.dominance	ANEW	0.13	0.06	–	0.05	–
ANEWEmo.mean.ang	ANEWEmo	0.12	0.09	–	0.08	0.32
ANEWEmo.mean.fear	ANEWEmo	0.14	0.1	–	0.09	0.38
ANEWEmo.mean.hap	ANEWEmo	0.15	0.08	–	0.08	–
ANEWEmo.mean.sad	ANEWEmo	0.16	0.12	0.04	0.11	0.38
GI.Milit	General Inquirer	-0.13	-0.12	-0.1	-0.14	–
GI.POLIT	General Inquirer	-0.13	-0.16	-0.1	-0.14	-0.3
GI.POWTOT	General Inquirer	-0.13	-0.14	-0.08	-0.16	-0.2
GI.SKLTOT	General Inquirer	-0.13	-0.13	-0.11	-0.15	-0.29
GI.SKLOTH	General Inquirer	-0.12	-0.11	-0.1	-0.13	-0.17
GI.COLL	General Inquirer	-0.11	-0.11	-0.07	-0.11	-0.11
GI.SKLPT	General Inquirer	-0.11	-0.1	-0.07	-0.11	-0.25
GI.Actv	General Inquirer	-0.1	-0.11	-0.05	-0.12	-0.15
GI.POWCON	General Inquirer	-0.1	-0.11	-0.1	-0.12	-0.1
GI.ECON	General Inquirer	-0.09	-0.13	-0.06	-0.11	-0.21
GI.Polit	General Inquirer	-0.09	-0.09	–	-0.1	-0.26
GI.Tool	General Inquirer	-0.09	-0.11	-0.05	-0.13	-0.29
GI.Exprs	General Inquirer	-0.08	-0.11	-0.07	-0.13	-0.34
GI.Comnobj	General Inquirer	-0.08	-0.06	–	-0.09	-0.16
GI.IAV	General Inquirer	-0.08	-0.07	–	-0.07	-0.1
GI.WLTTOT	General Inquirer	-0.08	-0.12	-0.05	-0.13	-0.2
GI.NATIONS	General Inquirer	-0.08	-0.07	-0.06	–	-0.16

Feature	Dictionary	Bipolar	Depression	ADHD	Anxiety	Stress
GI.Hostile	General Inquirer	-0.07	-0.07	-0.07	-0.07	0.1
GI.Vehicle	General Inquirer	-0.07	-0.11	-0.07	-0.11	-0.09
GI.Name	General Inquirer	-0.07	-0.08	-0.06	-0.05	-0.19
GI.DAV	General Inquirer	-0.07	-0.03	-0.04	-0.05	-0.06
GI.POWAREN	General Inquirer	-0.07	-0.08	-0.04	-0.06	-0.2
GI.POWCOOP	General Inquirer	-0.07	-0.07	-0.08	-0.08	-0.07
GI.POWPT	General Inquirer	-0.07	-0.06	-0.05	-0.08	-0.14
GI.WLTOTH	General Inquirer	-0.07	-0.12	-0.04	-0.13	-0.16
GI.Work	General Inquirer	-0.06	-0.03	–	-0.04	-0.09
GI.Ach	General Inquirer	-0.06	-0.04	-0.04	-0.05	-0.08
GI.Fetch	General Inquirer	-0.06	-0.04	–	-0.05	-0.08
GI.POWAPT	General Inquirer	-0.06	-0.1	-0.04	-0.11	-0.09
GI.TRANS	General Inquirer	-0.06	-0.04	–	-0.04	-0.14
GI.Power	General Inquirer	-0.05	-0.08	-0.05	-0.09	-0.22
GI.Sky	General Inquirer	-0.05	-0.03	–	-0.04	-0.15
GI.Object	General Inquirer	-0.05	-0.08	–	-0.09	-0.3
GI.Complt	General Inquirer	-0.05	–	-0.03	-0.03	–
GI.Begin	General Inquirer	-0.05	-0.08	-0.04	-0.08	-0.15
GI.Incr	General Inquirer	-0.05	–	-0.03	-0.06	–
GI.Exert	General Inquirer	-0.05	-0.05	–	-0.07	–
GI.Fall	General Inquirer	-0.05	–	–	-0.03	-0.05
GI.Space	General Inquirer	-0.05	-0.08	–	-0.07	-0.14
GI.POWGAIN	General Inquirer	-0.05	-0.04	-0.06	-0.05	–
GI.Econ	General Inquirer	-0.04	-0.07	–	-0.08	-0.09
GI.PLACE	General Inquirer	-0.04	-0.06	-0.04	-0.05	-0.11
GI.Land	General Inquirer	-0.04	-0.04	-0.04	-0.06	-0.16
GI.Change	General Inquirer	-0.04	-0.04	–	-0.03	–
GI.Move	General Inquirer	-0.04	–	–	–	–
GI.Travel	General Inquirer	-0.04	-0.06	–	-0.04	-0.1
GI.WLTPT	General Inquirer	-0.04	-0.04	–	-0.04	-0.09
GI.ENLGAIN	General Inquirer	-0.04	–	–	–	-0.1
GI.Virtue	General Inquirer	0.04	–	–	–	-0.22
GI.Ritual	General Inquirer	0.04	–	–	–	–
GI.HU	General Inquirer	0.04	0.04	–	0.04	–
GI.ANI	General Inquirer	0.04	–	–	–	-0.08
GI.Natpro	General Inquirer	0.04	–	–	0.05	0.07
GI.Abs	General Inquirer	0.04	–	–	–	-0.08
GI.DIM	General Inquirer	0.04	–	–	–	-0.16
GI.ANOMIE	General Inquirer	0.04	0.03	–	–	0.19
GI.NEGAFF	General Inquirer	0.04	0.03	–	–	0.22
GI.Ovrst	General Inquirer	0.05	0.03	0.04	–	0.15
GI.Relig	General Inquirer	0.05	–	–	–	-0.12
GI.Social	General Inquirer	0.05	–	–	–	0.12
GI.Need	General Inquirer	0.05	0.05	0.04	0.06	-0.08

Feature	Dictionary	Bipolar	Depression	ADHD	Anxiety	Stress
GI.Think	General Inquirer	0.05	–	0.05	–	–
GI.EVAL	General Inquirer	0.05	0.04	–	–	–
GI.Rel	General Inquirer	0.05	0.04	–	0.04	–
GI.IPadj	General Inquirer	0.05	0.04	0.03	0.04	–
GI.Neg	General Inquirer	0.06	0.04	–	–	0.46
GI.Affil	General Inquirer	0.06	0.05	–	0.06	0.14
GI.Subm	General Inquirer	0.06	0.06	0.07	0.04	0.1
GI.Psv	General Inquirer	0.06	0.05	0.04	0.05	0.29
GI.Arousal	General Inquirer	0.06	0.08	0.04	0.06	0.15
GI.Bodypt	General Inquirer	0.06	0.05	0.05	0.07	0.06
GI.Pos	General Inquirer	0.07	0.04	–	–	-0.21
GI.FOOD	General Inquirer	0.07	0.04	–	0.05	-0.08
GI.Pstv	General Inquirer	0.08	0.06	–	0.05	-0.14
GI.Weak	General Inquirer	0.08	0.05	0.03	0.05	0.39
GI.Pleasure	General Inquirer	0.08	0.04	0.04	0.04	-0.05
GI.Acad	General Inquirer	0.08	0.06	0.08	0.06	0.08
GI.Role	General Inquirer	0.08	0.08	0.05	0.09	0.07
GI.ABS	General Inquirer	0.08	0.04	–	–	0.08
GI.Vice	General Inquirer	0.09	0.07	–	0.05	0.31
GI.FREQ	General Inquirer	0.09	0.07	0.04	0.06	0.2
GI.TIME	General Inquirer	0.09	0.04	–	0.05	0.2
GI.Negate	General Inquirer	0.1	0.1	0.06	0.11	0.31
GI.IndAdj	General Inquirer	0.1	0.05	0.03	0.05	–
GI.AFFPT	General Inquirer	0.1	0.13	0.06	0.14	0.22
GI.Undrst	General Inquirer	0.11	0.08	0.05	0.09	0.12
GI.Food	General Inquirer	0.11	0.08	0.06	0.11	-0.08
GI.NOT	General Inquirer	0.11	0.1	0.06	0.11	0.31
GI.Nonadlt	General Inquirer	0.12	0.11	0.07	0.12	0.08
GI.Pain	General Inquirer	0.13	0.11	0.07	0.13	0.56
GI.EMOT	General Inquirer	0.13	0.1	0.06	0.1	0.49
GI.AFFOTH	General Inquirer	0.13	0.1	–	0.1	0.26
GI.WLBPSYC	General Inquirer	0.13	0.11	0.08	0.11	0.52
GI.WLBPT	General Inquirer	0.14	0.13	0.1	0.13	0.28
GI.PARTIC	General Inquirer	0.14	0.08	0.07	0.12	–
GI.Kin	General Inquirer	0.15	0.15	0.08	0.17	0.35
GI.AFFTOT	General Inquirer	0.15	0.15	0.07	0.17	0.31
GI.WLBPHYS	General Inquirer	0.17	0.15	0.1	0.21	0.31
GI.Female	General Inquirer	0.18	0.19	0.11	0.21	0.31
GI.WLBTOT	General Inquirer	0.22	0.21	0.14	0.24	0.61
GI.Self	General Inquirer	0.38	0.38	0.22	0.4	1.41
GI.Ngtv	General Inquirer	–	0.03	–	0.04	0.46
GI.Strng	General Inquirer	–	-0.07	-0.03	-0.09	-0.17
GI.Intrel	General Inquirer	–	0.03	–	–	0.08
GI.MALE	General Inquirer	–	0.06	–	0.07	0.3

Feature	Dictionary	Bipolar	Depression	ADHD	Anxiety	Stress
GI.Region	General Inquirer	–	-0.07	–	-0.04	-0.1
GI.Route	General Inquirer	–	-0.03	-0.05	-0.04	-0.06
GI.Bldgpt	General Inquirer	–	-0.04	–	-0.05	–
GI.Natobj	General Inquirer	–	0.04	0.05	–	–
GI.COM	General Inquirer	–	-0.03	–	-0.04	-0.18
GI.Means	General Inquirer	–	-0.05	–	-0.08	–
GI.Fail	General Inquirer	–	-0.05	–	-0.04	0.06
GI.Rise	General Inquirer	–	-0.04	-0.04	–	-0.09
GI.Know	General Inquirer	–	-0.04	–	–	-0.09
GI.Eval	General Inquirer	–	0.06	0.04	–	–
GI.Time	General Inquirer	–	0.03	–	–	0.23
GI.POWDOCT	General Inquirer	–	-0.04	–	-0.04	-0.11
GI.RSPGAIN	General Inquirer	–	0.03	–	–	–
GI.WLTTRAN	General Inquirer	–	-0.03	–	-0.04	-0.1
GI.WLBGAIN	General Inquirer	–	-0.04	–	-0.03	–
GI.ENLLOSS	General Inquirer	–	0.03	–	–	0.08
GI.TRNGAIN	General Inquirer	–	-0.04	–	-0.04	-0.06
GI.Doctr	General Inquirer	–	–	0.04	–	-0.12
GI.COLOR	General Inquirer	–	–	-0.04	–	-0.19
GI.ENLOTH	General Inquirer	–	–	0.06	–	-0.11
GI.ENLTOT	General Inquirer	–	–	0.05	–	-0.14
GI.MEANS	General Inquirer	–	–	0.04	–	–
GI.Aquatic	General Inquirer	–	–	–	-0.05	-0.12
GI.DIST	General Inquirer	–	–	–	-0.04	-0.09
GI.Yes	General Inquirer	–	–	–	0.04	-0.16
GI.Intrj	General Inquirer	–	–	–	0.04	-0.08
GI.POWLOSS	General Inquirer	–	–	–	-0.04	–
GI.TIMESP	General Inquirer	–	–	–	0.04	0.27
GI.Comform	General Inquirer	–	–	–	–	-0.13
GI.Goal	General Inquirer	–	–	–	–	-0.09
GI.Percv	General Inquirer	–	–	–	–	-0.11
GI.Comp	General Inquirer	–	–	–	–	-0.11
GI.Qual	General Inquirer	–	–	–	–	0.05
GI.Quan	General Inquirer	–	–	–	–	-0.18
GI.POS	General Inquirer	–	–	–	–	-0.09
GI.Dimn	General Inquirer	–	–	–	–	-0.04
GI.You	General Inquirer	–	–	–	–	-0.42
GI.POWENDS	General Inquirer	–	–	–	–	-0.1
GI.RCTETH	General Inquirer	–	–	–	–	0.07
GI.RCTREL	General Inquirer	–	–	–	–	-0.08
GI.RSPOTH	General Inquirer	–	–	–	–	0.06
GI.RSPTOT	General Inquirer	–	–	–	–	0.06
GI.WLBLOSS	General Inquirer	–	–	–	–	0.11
GI.ENLPT	General Inquirer	–	–	–	–	-0.06

Feature	Dictionary	Bipolar	Depression	ADHD	Anxiety	Stress
GI.SKLAS	General Inquirer	–	–	–	–	-0.13
GI.TRNLOSS	General Inquirer	–	–	–	–	0.07
GI.ENDS	General Inquirer	–	–	–	–	-0.17
GI.AUD	General Inquirer	–	–	–	–	-0.4
GI.POSAFF	General Inquirer	–	–	–	–	-0.16
GI.SURE	General Inquirer	–	–	–	–	-0.1
GI.IF	General Inquirer	–	–	–	–	-0.15
GI.FORM	General Inquirer	–	–	–	–	-0.12
GALC.Amusement	GALC	-0.19	-0.16	-0.13	-0.15	-0.34
GALC.Guilt	GALC	0.04	0.05	–	0.05	0.15
GALC.Sadness	GALC	0.04	–	–	–	0.1
GALC.Disgust	GALC	0.05	0.04	–	0.05	–
GALC.Gratitude	GALC	0.06	0.04	0.04	0.06	–
GALC.Longing	GALC	0.06	0.03	–	–	–
GALC.Anxiety	GALC	0.07	0.09	0.06	0.09	0.53
GALC.Lust	GALC	0.08	0.05	0.04	0.05	0.12
GALC.Fear	GALC	0.09	0.08	0.05	0.08	0.4
GALC.Feelinglove	GALC	0.13	0.11	–	0.1	0.09
GALC.Hope	GALC	–	0.04	–	0.04	-0.09
GALC.Irritation	GALC	–	0.03	–	0.04	–
GALC.Jealousy	GALC	–	0.03	–	–	–
GALC.Relief	GALC	–	0.03	0.04	0.05	0.09
GALC.Contentment	GALC	–	–	0.05	–	–
GALC.Dissatisfaction	GALC	–	–	0.04	–	–
GALC.Positive	GALC	–	–	–	-0.04	-0.23
GALC.Anger	GALC	–	–	–	–	0.15
GALC.Boredom	GALC	–	–	–	–	-0.12
GALC.Desperation	GALC	–	–	–	–	0.11
GALC.Happiness	GALC	–	–	–	–	0.06
GALC.Hatred	GALC	–	–	–	–	0.14
GALC.Humility	GALC	–	–	–	–	-0.04
GALC.Interest.Enthusiasm	GALC	–	–	–	–	-0.12
GALC.Shame	GALC	–	–	–	–	0.09
GALC.Tension.Stress	GALC	–	–	–	–	0.1
GALC.Negative	GALC	–	–	–	–	0.07
LIWC.Article	LIWC	-0.2	-0.2	-0.12	-0.22	-0.74
LIWC.Leisure	LIWC	-0.18	-0.16	-0.16	-0.17	-0.45
LIWC.Money	LIWC	-0.11	-0.13	-0.08	-0.12	-0.16
LIWC.See	LIWC	-0.08	-0.07	-0.08	-0.05	-0.44
LIWC.Achieve	LIWC	-0.07	-0.04	-0.05	-0.07	-0.19
LIWC.Power	LIWC	-0.07	-0.07	–	-0.09	0.08
LIWC.Death	LIWC	-0.06	-0.06	-0.06	-0.06	–
LIWC.Space	LIWC	-0.04	-0.08	–	-0.08	-0.15
LIWC.Nonflu	LIWC	0.04	0.04	–	–	–

Feature	Dictionary	Bipolar	Depression	ADHD	Anxiety	Stress
LIWC.Affiliation	LIWC	0.05	0.07	–	0.07	0.17
LIWC.Relig	LIWC	0.05	0.03	–	–	-0.13
LIWC.Sad	LIWC	0.06	0.06	–	0.05	0.34
LIWC.Tentat	LIWC	0.06	0.05	0.07	0.07	-0.12
LIWC.Certain	LIWC	0.06	0.05	–	0.06	–
LIWC.Differ	LIWC	0.06	0.05	0.06	0.07	–
LIWC.Negate	LIWC	0.07	0.07	–	0.09	0.23
LIWC.Sexual	LIWC	0.07	0.05	–	0.04	0.05
LIWC.Adj	LIWC	0.08	0.05	0.04	0.07	-0.15
LIWC.Time	LIWC	0.08	0.08	–	0.05	0.37
LIWC.Posemo	LIWC	0.09	0.05	–	0.06	-0.39
LIWC.Ipron	LIWC	0.1	0.08	0.09	0.1	0.09
LIWC.Focuspast	LIWC	0.1	0.13	0.04	0.14	0.3
LIWC.Shehe	LIWC	0.11	0.15	0.04	0.16	0.45
LIWC.Adverb	LIWC	0.11	0.17	0.09	0.15	0.25
LIWC.Body	LIWC	0.12	0.1	0.08	0.11	0.12
LIWC.Conj	LIWC	0.13	0.12	0.07	0.12	0.28
LIWC.Insight	LIWC	0.13	0.12	0.13	0.14	0.3
LIWC.Ingest	LIWC	0.13	0.1	0.08	0.14	-0.12
LIWC.Anx	LIWC	0.14	0.14	0.09	0.15	0.68
LIWC.Home	LIWC	0.15	0.09	0.05	0.1	0.32
LIWC.Focuspresent	LIWC	0.17	0.15	0.11	0.18	0.39
LIWC.Family	LIWC	0.18	0.18	0.09	0.2	0.37
LIWC.Feel	LIWC	0.18	0.14	0.11	0.15	0.42
LIWC.Female	LIWC	0.19	0.2	0.11	0.22	0.33
LIWC.Auxverb	LIWC	0.2	0.19	0.14	0.22	0.4
LIWC.Verb	LIWC	0.24	0.25	0.15	0.28	0.57
LIWC.Health	LIWC	0.26	0.24	0.2	0.28	0.58
LIWC.I	LIWC	0.38	0.39	0.22	0.4	1.42
LIWC.Interrog	LIWC	–	0.05	0.05	–	0.22
LIWC.Number	LIWC	–	-0.04	-0.04	-0.07	-0.18
LIWC.Friend	LIWC	–	0.07	–	0.07	0.12
LIWC.Male	LIWC	–	0.07	–	0.09	0.32
LIWC.Prep	LIWC	–	–	0.06	–	0.08
LIWC.Anger	LIWC	–	–	-0.04	–	0.25
LIWC.Cause	LIWC	–	–	0.06	–	–
LIWC.Reward	LIWC	–	–	-0.05	-0.04	-0.28
LIWC.Work	LIWC	–	–	0.05	-0.06	-0.14
LIWC.You	LIWC	–	–	–	–	-0.61
LIWC.They	LIWC	–	–	–	–	-0.28
LIWC.Compare	LIWC	–	–	–	–	-0.31
LIWC.Quant	LIWC	–	–	–	–	-0.17
LIWC.Discrep	LIWC	–	–	–	–	-0.06
LIWC.Hear	LIWC	–	–	–	–	-0.06

Feature	Dictionary	Bipolar	Depression	ADHD	Anxiety	Stress
LIWC.Risk	LIWC	–	–	–	–	0.27
LIWC.Focusfuture	LIWC	–	–	–	–	-0.08
LIWC.Motion	LIWC	–	–	–	–	0.06
LIWC.Netspeak	LIWC	–	–	–	–	-0.13
LIWC.Assent	LIWC	–	–	–	–	-0.19
EmoLex.surprise	NRC EmoLex	0.04	–	–	–	–
EmoLex.anticipation	NRC EmoLex	0.05	0.03	–	–	–
EmoLex.sadness	NRC EmoLex	0.05	0.06	–	0.06	0.61
EmoLex.trust	NRC EmoLex	0.06	–	–	–	-0.18
EmoLex.disgust	NRC EmoLex	0.08	0.08	–	0.07	0.34
EmoLex.positive	NRC EmoLex	0.08	–	–	–	-0.35
EmoLex.joy	NRC EmoLex	0.15	0.1	–	0.08	-0.16
EmoLex.anger	NRC EmoLex	–	–	-0.03	–	0.49
EmoLex.fear	NRC EmoLex	–	–	-0.04	–	0.52
EmoLex.negative	NRC EmoLex	–	–	–	–	0.6
SenticNet.neg	SenticNet	0.08	0.07	–	0.06	0.38
SenticNet.pos	SenticNet	0.09	0.05	0.06	0.06	-0.17

Table S4: Comparison of how often our models rely on words from LIWC categories to make their decisions, according to AGRAD. Numbers represent the percentage of available LIWC words each model selected in the top 10 AGRAD explanations for the entire test set.

Category	Model Type	Bipolar	Depression	ADHD	Anxiety	Stress
Function Words	MentalRoBERTa	37.99	38.51	38.66	37.48	45.62
Pronouns	MentalRoBERTa	10.1	11.97	11.55	10.88	15.52
Personal Pronouns	MentalRoBERTa	5.71	7.97	6.85	7.14	11.02
I	MentalRoBERTa	2.65	4.26	2.73	3.72	6.2
You	MentalRoBERTa	1.28	1.26	1.75	1.13	1.65
Affective Processes	MentalRoBERTa	6.57	5.45	5.85	6.02	7.09
Negative Emotions	MentalRoBERTa	2.28	1.74	1.81	2.05	3.08
Social	MentalRoBERTa	7.72	9.67	9.14	9.12	10.69
Cognitive Processes	MentalRoBERTa	13.23	11.86	12.06	12.27	12.7
Time Orientation	MentalRoBERTa	13.66	13.84	13.03	13.84	16.04
Relativity	MentalRoBERTa	11.75	11.81	12.74	12.04	14.15
Function Words	+Emotion	37.39	38.67	38.93	38.16	44.79
Pronouns	+Emotion	10.53	11.69	11.19	11.21	15.74
Personal Pronouns	+Emotion	6.31	7.24	7.06	6.92	10.92
I	+Emotion	2.83	3.89	3.39	3.68	6.76
You	+Emotion	1.32	1.12	1.37	1.26	1.19
Affective Processes	+Emotion	6.03	5.6	5.88	6.08	7.51
Negative Emotions	+Emotion	1.93	1.91	1.85	2.21	3.45
Social	+Emotion	8.55	8.52	9.49	8.56	9.68
Cognitive Processes	+Emotion	12.75	12.26	11.67	13.45	13.03

Table S5. SP-LIME feature ablation results for General Linguistic Features (GLFs)

MHS	ADHD	Anxiety	Bipolar	Depression	Stress
Cohesion	3.00	7.16	4.94	6.64	4.04
Lexical	3.13	6.78	5.70	5.67	2.28
Morphosyntatic	2.96	7.07	4.68	6.32	2.94
Stylistic	3.25	6.57	4.84	6.08	5.52
Readability	2.92	4.59	4.66	2.81	2.60

Category	Model Type	ADHD	Anxiety	Bipolar	Depres.	Stress
Time Orientation	+Emotion	13.11	13.3	13.16	14.09	15.15
Relativity	+Emotion	11.87	12.23	12.56	12.45	13.7
Function Words	+Personality	38.88	39.57	37.96	40.12	44.75
Pronouns	+Personality	10.99	12.32	10.96	12.8	16.06
Personal Pronouns	+Personality	6.57	8.04	6.69	8.47	10.97
I	+Personality	2.82	4.22	3.46	5.1	6.55
You	+Personality	1.85	1.17	1.22	1.24	1.34
Affective Processes	+Personality	6.12	5.56	5.97	5.7	7.96
Negative Emotions	+Personality	1.85	1.63	1.93	1.98	4.1
Social	+Personality	8.27	9.59	8.95	8.8	10.43
Cognitive Processes	+Personality	12.84	12.06	12.77	12.54	13.52
Time Orientation	+Personality	13.46	13.11	13.18	13.53	16.2
Relativity	+Personality	12.17	11.92	12.44	11.87	13.81
Function Words	+Emotion+Personality	38.32	38.24	38.61	37.57	44.96
Pronouns	+Emotion+Personality	10.61	11.13	10.82	10.77	14.12
Personal Pronouns	+Emotion+Personality	6.17	7.26	6.6	6.75	8.98
I	+Emotion+Personality	2.63	3.91	3.38	3.18	4.72
You	+Emotion+Personality	1.49	1.01	1.17	1.26	1.05
Affective Processes	+Emotion+Personality	5.92	5.77	5.84	5.74	5.95
Negative Emotions	+Emotion+Personality	1.93	1.99	2.02	2.05	2.61
Social	+Emotion+Personality	8.28	8.7	8.63	9.25	10.39
Cognitive Processes	+Emotion+Personality	13.64	12.81	13.09	12.17	13.74
Time Orientation	+Emotion+Personality	13.8	13.14	13.72	13.78	15.97
Relativity	+Emotion+Personality	12.05	12.14	11.86	12.21	14.76
